# Asymmetric Cyclic Controlled Quantum Teleportation via Multiple-Qubit Entangled State in a Noisy Environment

**DOI:** 10.3390/e26121108

**Published:** 2024-12-18

**Authors:** Hanxuan Zhou

**Affiliations:** Information Science and Technology College, Dalian Maritime University, Dalian 116026, China; zhouhx@dlmu.edu.cn

**Keywords:** asymmetric, quantum teleportation, noisy environment, security

## Abstract

In this paper, by using eleven entangled quantum states as a quantum channel, we propose a cyclic and asymmetric novel protocol for four participants in which both Alice and Bob can transmit two-qubit states, and Charlie can transmit three-qubit states with the assistance of the supervisor David, who provides a guarantee for communication security. This protocol is based on GHZ state measurement (GHZ), single-qubit measurement (SM), and unitary operations (UO) to implement the communication task. The analysis demonstrates that the success probability of the proposed protocol can reach 100%. Furthermore, considering that in actual production environments, it is difficult to avoid the occurrence of noise in quantum channels, this paper also analyzes the changes in fidelity in four types of noisy scenarios: bit-flip noise, phase-flip noise, bit-phase-flip noise, and depolarizing noise. Showing that communication quality only depends on the amplitude parameters of the initial state and decoherence rate. Additionally, we give a comparison with previous similar schemes in terms of achieved method and intrinsic efficiency, which illustrates the superiority of our protocol. Finally, in response to the vulnerability of quantum channels to external attacks, a security analysis was conducted, and corresponding defensive measures were proposed.

## 1. Introduction

One of the most peculiar applications of quantum information is quantum teleportation, which, in principle, allows the faithful transfer of an unknown quantum state from one particle to another particle without physical transmission of the object itself. Since the quantum teleportation protocol was proposed by Bennett et al. in 1993 [1], the related research has received wide attention from theoretical and experimental researchers in recent years for its important applications in quantum computation and quantum communication. Subsequently, some kinds of schemes of quantum teleportation mainly based on point-to-point unidirectional mode have been presented [2,3,4,5,6,7,8]. Alternatively, some researchers have found that increasing security by adding a supervisor leads to more efficient transmission [9,10,11,12,13,14]. In 2013, Zha et al. innovatively proposed a bidirectional control protocol with a five-qubit cluster state; transmission further develops from a point-to-point unidirectional mode to a bidirectional mode [15]. In 2014, Duan et al. realized quantum teleportation based on a six-qubit entangled state [16]. Further, several kinds of bidirectional controlled joint remote state preparation [17,18] and bidirectional controlled hybrid communication schemes [19,20] have been explored.

When considering more participants existed in quantum teleportation protocol, cyclic teleportation was proposed by Chen et al. in 2017 [21], which can be considered as a further extension of bidirectional teleportation. In Chen’s scheme, Alice, Bob, and Charlie cyclically teleport three arbitrary single-qubit states amongst themselves, which are in their respective possessions, using a six-qubit maximally entangled state as a quantum channel. In 2018, Sang et al. [22] designed a cyclic teleportation scheme with a control party. Similar to the above schemes, Wang et al. studied the four-participant cyclic controlled remote state preparation scheme of a single-qubit state [23]. In 2020, the double-direction cyclic controlled communication of a single-qubit state via a thirteen-qubit entangled state was put forward by Sun et al. [24].

Zhang and Duan proposed an asymmetric bidirectional quantum teleportation protocol in 2015 [25], which utilized a maximally seven-qubit entangled state as a quantum channel. In 2017, another asymmetric teleportation protocol was created by Choudhury et al. [26]. Moreover, cyclic-controlled protocols were proposed based on asymmetric teleportation [27,28]. Such communication is under the control of a supervisor and has the advantage of avoiding the attack of eavesdroppers [27].

To teleport more particles among three senders with high efficiency, we propose a novel protocol on the basis of a combination of the traditional asymmetric model and the cyclic model of teleportation, where the quantum channel is eleven-qubit entangled states. With the help of controller David, the senders Alice, Bob, and Charlie can exchange their desired quantum state at the same time; by following the protocol, the states are transferred with unit probability. This is in contrast with the probabilistic teleportation schemes, in which state transfers only have a certain probability of success. Additionally, unlike most protocols that only transmit a small number of particles and communicate between two parties, we innovatively propose a quantum channel constructed with very few entangled quantum states, which can transmit more quantum states and achieve multi-party cyclic transmission. Compared with symmetric communication, our asymmetric protocol can reduce unnecessary quantum state reconstruction, thereby saving the consumption of quantum resources and making the communication method more flexible. Moreover, due to the existence of the controller in our protocol, it can choose whether to notify the communicating party of the measurement results to achieve the desired control effect. From a communication engineering perspective, for each of the three neighboring observers, they can transmit different quantum states simultaneously, which is more suitable for future quantum communication networks. Furthermore, in reality, noises are inevitably introduced when teleporting a quantum state [29,30,31,32,33]. In order to implement the protocol in practice, we also consider the effect of the noise environment on the teleportation. Finally, we conducted a security analysis and proposed corresponding measures for the vulnerability of quantum channels to external attacks.

The rest of this article is organized as follows: Section 2 details the establishment of the quantum channel for the proposed protocol and the description of the protocol. In Section 3, we calculate the fidelity of the proposed protocol under four noise channels and then analyze and compare them through MATLAB 2021a simulations. In Section 4, we first perform an efficiency analysis and make comparisons with other similar proposed protocols, and then we discuss the security of the proposed scheme. Finally, Section 5 draws a conclusion.

## 2. The Scheme of ACCQT Protocol

### 2.1. Construction of the Quantum Channel

**Figure 1:**
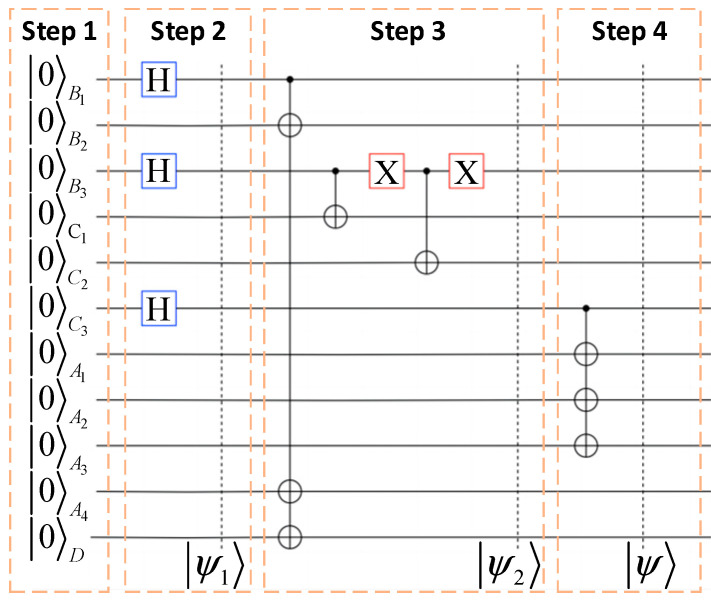
Quantum circuit to prepare an entangled channel. The details of Step 1 to Step 4 in the figure can be found, respectively, in the main text.

This quantum channel can not only be theoretically proposed but also constructed. The feasibility of a proposed quantum channel is depicted in Figure 1.

In the ideal environment, the steps involved in creating a channel are described as follows:

***Step 1***. Preparing eleven qubits in the state |0⟩ as the initial state:

(1) $${|\Psi_{0}\rangle }_{B_{1}B_{2}B_{3}C_{1}C_{2}C_{3}A_{1}A_{2}A_{3}A_{4}D}=|0\rangle_{B_{1}B_{2}B_{3}C_{1}C_{2}C_{3}A_{1}A_{2}A_{3}A_{4}D}^{⊗11}$$

***Step 2***. Applying three Hadamard gate operations to the qubits *B*_1_, *B*_3_, and *C*_3_, respectively, then the above input state |Ψ_0_⟩ can be transformed as:

(2) $${|\Psi_{1}\rangle }_{B_{1}B_{2}B_{3}C_{1}C_{2}C_{3}A_{1}A_{2}A_{3}A_{4}D}=|+\rangle_{B_{1}}⊗|0\rangle_{B_{2}}⊗|+\rangle_{B_{3}}⊗|0\rangle_{C_{1}}⊗|0\rangle_{C_{2}}⊗|+\rangle_{C_{3}}⊗|0\rangle_{A_{1}A_{2}A_{3}A_{4}D}^{⊗5}$$

***Step 3***. This step implements CNOT gate operations on qubits (*B*_1_, *B*_2_), (*B*_1_, *A*_4_), (*B*_1_, *D*), and (*B*_3_, *C*_1_) and (*B*_3_, *C*_2_), respectively, subsequently applying the Pauli-X gate operation twice on qubit *B*_3_. Then, the state will convert into |Ψ_2_⟩, that is shown as follows:

(3) $$\begin{array}{l} |\Psi_{2}\rangle_{B_{1}B_{2}B_{3}C_{1}C_{2}C_{3}A_{1}A_{2}A_{3}A_{4}D}=\frac{1}{2\sqrt{2}}(|00001000000\rangle +|00001100000\rangle +|00110000000\rangle +|00110100000\rangle \\ +|11001000011\rangle +|11001100011\rangle +|111100000011\rangle +|11110100011\rangle {)}_{B_{1}B_{2}B_{3}C_{1}C_{2}C_{3}A_{1}A_{2}A_{3}A_{4}D} \end{array}$$

where the CNOT gate is applied to two qubits, treat the first qubit as the control qubit and the second qubit as the target qubit. The Pauli-X gate is a single-qubit gate operation that performs a flip operation on a quantum bit, transforming the state from |0⟩ to |1⟩. They can be represented as follows:

(4) $$\mathrm{CNOT}=\left[\begin{array}{cc} \begin{array}{cc} 1 & 0\\ 0 & 1 \end{array} & \begin{array}{cc} 0 & 0\\ 0 & 0 \end{array}\\ \begin{array}{cc} 0 & 0\\ 0 & 0 \end{array} & \begin{array}{cc} 0 & 1\\ 1 & 0 \end{array} \end{array}\right],\;X=\left[\begin{array}{cc} 0 & 1\\ 1 & 0 \end{array}\right]$$

***Step 4***. Then applying CNOT gate operations on *A*_1_, *A*_2_, and *A*_3_ as target qubits and qubit *C*_3_ as a controlled qubit is performed. Finally, we can obtain the final constructed quantum channel |Ψ⟩ through the above operations, and it can be described as:

(5) $$\begin{array}{l} {|\psi \rangle }_{B_{1}B_{2}B_{3}C_{1}C_{2}C_{3}A_{1}A_{2}A_{3}A_{4}D}=\frac{1}{2\sqrt{2}}(|00001000000\rangle +|00001111100\rangle +|00110000000\rangle +|00110111100\rangle \\ +|11001000011\rangle +|11001111111\rangle +|11110000011\rangle +|11110111111\rangle {)}_{B_{1}B_{2}B_{3}C_{1}C_{2}C_{3}A_{1}A_{2}A_{3}A_{4}D} \end{array}$$

### 2.2. Description of the Proposed Protocol

**Figure 2:**
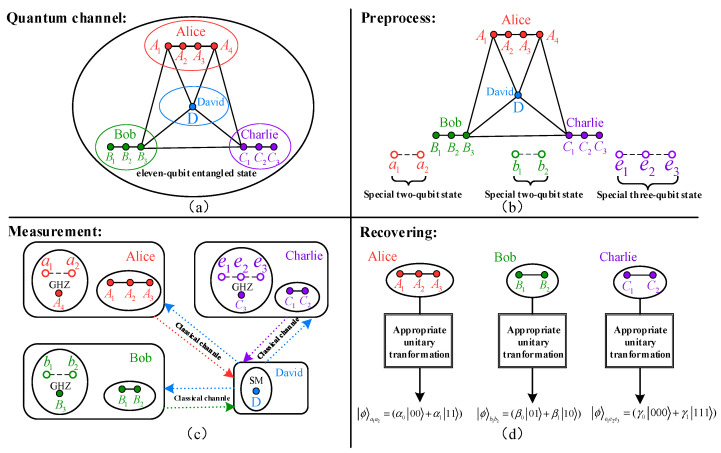
The schematic diagram of asymmetric cyclic controlled quantum teleportation. (**a**) The solid circle represents the qubits on the quantum channel; (**b**) the hollow circle represents the qubits to be transmitted between Alice, Bob, and Charlie; (**c**) SM denotes single-qubit state measurement, and GHZ denotes GHZ-state measurement. Among them, Alice and Bob measured the three-qubit GHZ state, and Charlie measured the four-qubit GHZ state and sent it to David through the classical channel. David measured the single-qubit and broadcast the measurement results through the classical channel; (**d**) Alice, Bob, and Charlie performed the corresponding unitary operation.

Based on the quantum channel we prepared, in this section, we give a detailed description of the proposed scheme, as shown in Figure 2.

The proposed protocol is described as follows: Suppose Alice holds an unknown two-qubit state ${\left|\phi \right\rangle }_{{a_{1}a}_{2}}$, which is denoted by:

(6) $${\left|\phi \right\rangle }_{a_{1}a_{2}}=(\alpha_{0}|00\rangle +\alpha_{1}|11\rangle )$$

$\alpha_{0}$,$\alpha_{1}$ are complex numbers with ${{|\alpha }_{0}|}^{2}+{{|\alpha }_{1}|}^{2}=1$. The state ${\;|\phi \rangle }_{{a_{1}a}_{2}}\;$is to be teleported to Bob. Simultaneously, Bob wants to teleport an unknown two-qubit state to Charlie, and David wants to teleport an unknown three-qubit state to Alice, which can be expressed as:

(7) $${\left|\phi \right\rangle }_{b_{1}b_{2}}=({𝛽}_{0}|01\rangle +{𝛽}_{1}|10\rangle )$$

(8) $${\left|\phi \right\rangle }_{e_{1}e_{2}e_{3}}=({𝛾}_{0}|000\rangle +{𝛾}_{1}|111\rangle )$$

where *β*_0_, *β*_1_, *γ*_0_, *γ*_1_ are complex numbers with ${{|\beta }_{0}|}^{2}+|{\beta_{1}|}^{2}=1$ and ${{|\gamma }_{0}|}^{2}+|{\gamma_{1}|}^{2}=1$. To effectively implement the asymmetric cyclic controlled quantum teleportation protocol, we need to perform the following six steps:

***Step 1***. **Define the holding of qubit information**

Supposing that Alice, Bob, Charlie, and supervisor David share the eleven-qubit entangled state |Ψ⟩, where Alice holds the qubits *A*_1_, *A*_2_, *A*_3_, and *A*_4_; Bob possesses the qubits *B*_1_, *B*_2_, and *B*_3_; Charlie possesses the qubits *C*_1_, *C*_2_, and *C*_3_; and qubit D is being held by David. Consequently, the overall system can be described as follows:

(9) $$|\Psi \rangle_{a_{1}a_{2}b_{1}b_{2}e_{1}e_{2}e_{3}B_{1}B_{2}B_{3}C_{1}C_{2}C_{3}A_{1}A_{2}A_{3}A_{4}D}=|\phi \rangle_{a_{1}a_{2}}⊗|\phi \rangle_{b_{1}b_{2}}⊗|\phi \rangle_{e_{1}e_{2}e_{3}}⊗|\Psi \rangle_{B_{1}B_{2}B_{3}C_{1}C_{2}C_{3}A_{1}A_{2}A_{3}A_{4}D}$$

***Step 2***. **The detailed measurement for Alice**

Afterward, Alice conducts a three-qubit GHZ state measurement (GSM) on qubits (*a*_1_, *a*_2_, *A*_4_) and communicates her measurement results to Bob, where she can choose randomly one of eight GHZ states |*δ*^+^⟩a_1_a_2_A_4_, |*δ*^−^⟩a_1_a_2_A_4_, |*η*^+^⟩a_1_a_2_A_4_, |*η*^−^⟩a_1_a_2_A_4_, |*w*^−^⟩a_1_a_2_A_4_, |*w*^+^⟩a_1_a_2_A_4_, |μ^−^⟩a_1_a_2_A_4_, |μ^+^⟩a_1_a_2_A_4_ as the measurement basis. The eight distinct three-qubit GHZ states are defined as follows:

(10) $$\begin{array}{l} \begin{array}{l} \left|\delta^{+}\rangle_{a_{1}a_{2}A_{4}}=\frac{1}{\sqrt{2}}(|000\rangle +|111\rangle ),|\delta^{-}\rangle_{a_{1}a_{2}A_{4}}=\frac{1}{\sqrt{2}}(|000\rangle -|111\rangle \right)\\ \left|\eta^{+}\rangle_{a_{1}a_{2}A_{4}}=\frac{1}{\sqrt{2}}(|001\rangle +|110\rangle ),|\eta^{-}\rangle_{a_{1}a_{2}A_{4}}=\frac{1}{\sqrt{2}}(|001\rangle -|110\rangle \right) \end{array}\\ \begin{array}{l} \left|\omega^{+}\rangle_{a_{1}a_{2}A_{4}}=\frac{1}{\sqrt{2}}(|010\rangle +|101\rangle ),|\omega^{-}\rangle_{a_{1}a_{2}A_{4}}=\frac{1}{\sqrt{2}}(|010\rangle -|101\rangle \right)\\ \left|\mu^{+}\rangle_{a_{1}a_{2}A_{4}}=\frac{1}{\sqrt{2}}(|100\rangle +|011\rangle ),|\mu^{-}\rangle_{a_{1}a_{2}A_{4}}=\frac{1}{\sqrt{2}}(|100\rangle -|011\rangle \right) \end{array} \end{array}$$

In order to realize the asymmetric cyclic control teleportation protocol, Alice must perform a measurement on her three qubits, *a*_1_, *a*_2_, and *A*_4_, on the basis that is given in Equation (10). With this basis, the quantum system can be expressed as:

(11) $$\begin{array}{l} |\Psi \rangle_{b_{1}b_{2}e_{1}e_{2}e_{3}a_{1}a_{2}A_{4}B_{1}B_{2}B_{3}C_{1}C_{2}C_{3}A_{1}A_{2}A_{3}D}=\frac{1}{2}|\phi \rangle_{b_{1}b_{2}}⊗|\phi \rangle_{e_{1}e_{2}e_{3}}⊗{\{|\delta^{+}\rangle }_{a_{1}a_{2}A_{4}}\\ ⊗[\alpha_{0}(|0000100000\rangle +|0000111110\rangle +|0011000000\rangle +|0011011110\rangle )\\ +\alpha_{1}(|1100100001\rangle +|1100111111\rangle +|1111000001\rangle +|1111011111\rangle )]\\ +|\delta^{-}\rangle_{a_{1}a_{2}A_{4}}⊗[\alpha_{0}(|0000100000\rangle +|0000111110\rangle +|0011000000\rangle +|0011011110\rangle )\\ -\alpha_{1}(|1100100001\rangle +|1100111111\rangle +|1111000001\rangle +|1111011111\rangle )]\\ +|\eta^{+}\rangle_{a_{1}a_{2}A_{4}}⊗[\alpha_{0}(|1100100001\rangle +|1100111111\rangle +|1111000001\rangle +|1111011111\rangle )\\ +\alpha_{1}(|0000100000\rangle +|0000111110\rangle +|0011000000\rangle +|0011011110\rangle )]\\ +|\eta^{-}\rangle_{a_{1}a_{2}A_{4}}⊗[\alpha_{0}(|1100100001\rangle +|1100111111\rangle +|1111000001\rangle +|1111011111\rangle )\\ -\alpha_{1}(|0000100000\rangle +|0000111110\rangle +|0011000000\rangle +|0011011110\rangle ){]}_{B_{1}B_{2}B_{3}C_{1}C_{2}C_{3}A_{1}A_{2}A_{3}D}\} \end{array}$$

Suppose Alice obtains |*δ*^+^⟩a_1_a_2_A_4_ as a measurement outcome; in this case, she will share it through the classical channel, and the state of the remaining particles (*B*_1_, *B*_2_, *B*_3_, *B*_1_, *C*_2_, *C*_3_, *A*_1_, *A*_2_, *A*_3_, *D*) belonging to Alice, Bob, Charlie, and David will be in the state:

(12) $$\begin{array}{l} |\Psi \rangle_{b_{1}b_{2}e_{1}e_{2}e_{3}B_{1}B_{2}B_{3}C_{1}C_{2}C_{3}A_{1}A_{2}A_{3}D}^{\delta^{+}}=\frac{1}{2}|\phi \rangle_{b_{1}b_{2}}⊗|\phi \rangle_{e_{1}e_{2}e_{3}}\\ ⊗[\alpha_{0}(|0000100000\rangle +|0000111110\rangle +|0011000000\rangle +|0011011110\rangle {)}_{B_{1}B_{2}B_{3}C_{1}C_{2}C_{3}A_{1}A_{2}A_{3}D}\\ +\alpha_{1}(|1100100001\rangle +|1100111111\rangle +|1111000001\rangle +|1111011111\rangle {)}_{B_{1}B_{2}B_{3}C_{1}C_{2}C_{3}A_{1}A_{2}A_{3}D}] \end{array}$$

***Step 3.*** **The detailed measurement for Bob**

Then, Bob, instructed by Alice, performs a three-qubit GHZ state measurement (GSM) on qubits (*b*_1_, *b*_2_, *B*_3_) and communicates his measurement outcomes to Charlie. Bob’s measurement basis is composed of the following eight orthogonal states:

(13) $$\begin{array}{l} \begin{array}{l} \left|\delta^{+}\rangle_{b_{1}b_{2}B_{3}}=\frac{1}{\sqrt{2}}(|000\rangle +|111\rangle ),|\delta^{-}\rangle_{b_{1}b_{2}B_{3}}=\frac{1}{\sqrt{2}}(|000\rangle -|111\rangle \right)\\ \left|\eta^{+}\rangle_{b_{1}b_{2}B_{3}}=\frac{1}{\sqrt{2}}(|001\rangle +|110\rangle ),|\eta^{-}\rangle_{b_{1}b_{2}B_{3}}=\frac{1}{\sqrt{2}}(|001\rangle -|110\rangle \right) \end{array}\\ \begin{array}{l} \left|\omega^{+}\rangle_{b_{1}b_{2}B_{3}}=\frac{1}{\sqrt{2}}(|010\rangle +|101\rangle ),|\omega^{-}\rangle_{b_{1}b_{2}B_{3}}=\frac{1}{\sqrt{2}}(|010\rangle -|101\rangle \right)\\ \left|\mu^{+}\rangle_{b_{1}b_{2}B_{3}}=\frac{1}{\sqrt{2}}(|011\rangle +|100\rangle ),|\mu^{-}\rangle_{b_{1}b_{2}B_{3}}=\frac{1}{\sqrt{2}}(|011\rangle -|100\rangle \right) \end{array} \end{array}$$

With this basis, after completing the measurement on his three-qubit state, Bob transmits the measurement outcome to the controller, David. Suppose Bob’s measurement outcome is |*ω*^+^⟩*b_1_b_2_B_3_*. Then the state of the remaining particles (*B*_1_, *B*_2_, *C*_1_, *C*_2_, *C*_3_, *A*_1_, *A*_2_, *A*_3_, *D*) owned by Alice, Bob, Charlie, and David will be collapsed as:

(14) $$\begin{array}{l} |\Psi \rangle_{e_{1}e_{2}e_{3}B_{1}B_{2}C_{1}C_{2}C_{3}A_{1}A_{2}A_{3}D}^{\delta^{+},\omega^{+}}=\frac{1}{\sqrt{2}}⊗|\phi \rangle_{e_{1}e_{2}e_{3}}\\ ⊗[\alpha_{0}\beta_{0}(|000100000\rangle +|000111110\rangle )+\alpha_{0}\beta_{1}(|001000000\rangle +|001011110\rangle {)}_{B_{1}B_{2}C_{1}C_{2}C_{3}A_{1}A_{2}A_{3}D}\\ +\alpha_{1}\beta_{0}(|110100001\rangle +|110111111\rangle )+\alpha_{1}\beta_{1}(|111000001\rangle +|111011111\rangle {)}_{B_{1}B_{2}C_{1}C_{2}C_{3}A_{1}A_{2}A_{3}D}] \end{array}$$

***Step 4.*** **The detailed measurement for Charlie**

After that, under Bob’s instruction, Charlie proceeds to perform a four-qubit GHZ state measurement on qubit (*e*_1_, *e*_2_, *e*_3_, *C*_3_) and shares the measurement outcomes with Alice through a classical channel. Charlie’s measurement basis comprises the following orthogonal states:

(15) $$\begin{array}{l} |{\varepsilon_{1}}^{+}\rangle_{e_{1}e_{2}e_{3}C_{3}}=\frac{|0000\rangle +|1111\rangle }{\sqrt{2}},|{\varepsilon_{1}}^{-}\rangle_{e_{1}e_{2}e_{3}C_{3}}=\frac{|0000\rangle -|1111\rangle }{\sqrt{2}}\\ |{l_{1}}^{+}\rangle_{e_{1}e_{2}e_{3}C_{3}}=\frac{|0001\rangle +|1110\rangle }{\sqrt{2}},|{l_{1}}^{-}\rangle_{e_{1}e_{2}e_{3}C_{3}}=\frac{|0001\rangle -|1110\rangle }{\sqrt{2}}\\ |{\varepsilon_{2}}^{+}\rangle_{e_{1}e_{2}e_{3}C_{3}}=\frac{|0010\rangle +|1101\rangle }{\sqrt{2}},|{\varepsilon_{2}}^{-}\rangle_{e_{1}e_{2}e_{3}C_{3}}=\frac{|0010\rangle -|1101\rangle }{\sqrt{2}}\\ |{l_{2}}^{+}\rangle_{e_{1}e_{2}e_{3}C_{3}}=\frac{|0100\rangle +|1011\rangle }{\sqrt{2}},|{l_{2}}^{-}\rangle_{e_{1}e_{2}e_{3}C_{3}}=\frac{|0100\rangle -|1011\rangle }{\sqrt{2}}\\ |{\varepsilon_{3}}^{+}\rangle_{e_{1}e_{2}e_{3}C_{3}}=\frac{|1000\rangle +|0111\rangle }{\sqrt{2}},|{\varepsilon_{3}}^{-}\rangle_{e_{1}e_{2}e_{3}C_{3}}=\frac{|1000\rangle -|0111\rangle }{\sqrt{2}}\\ |{l_{3}}^{+}\rangle_{e_{1}e_{2}e_{3}C_{3}}=\frac{|0011\rangle +|1100\rangle }{\sqrt{2}},|{l_{3}}^{-}\rangle_{e_{1}e_{2}e_{3}C_{3}}=\frac{|0011\rangle -|1100\rangle }{\sqrt{2}}\\ |{\varepsilon_{4}}^{+}\rangle_{e_{1}e_{2}e_{3}C_{3}}=\frac{|0110\rangle +|1001\rangle }{\sqrt{2}},|{\varepsilon_{4}}^{-}\rangle_{e_{1}e_{2}e_{3}C_{3}}=\frac{|0110\rangle -|1001\rangle }{\sqrt{2}}\\ |{l_{4}}^{+}\rangle_{e_{1}e_{2}e_{3}C_{3}}=\frac{|1100\rangle +|0011\rangle }{\sqrt{2}},|{l_{4}}^{-}\rangle_{e_{1}e_{2}e_{3}C_{3}}=\frac{|1100\rangle -|0011\rangle }{\sqrt{2}} \end{array}$$

With the above orthogonal state’s measurement basis, suppose Charlie’s measurement outcome is |*ε*_1_^+^⟩*e_1_e_2_e_3_C_3_*. Then, the state of the recombining particles (*B*_1_, *B*_2_, *C*_1_, *C*_2_, *A*_1_, *A*_2_, *A*_3_, *D*) owned by Alice, Bob, Charlie, and David will be collapsed into the following:

(16) $$\begin{array}{l} |\Psi \rangle_{B_{1}B_{2}C_{1}C_{2}A_{1}A_{2}A_{3}D}^{\delta^{+},\omega^{+},{\varepsilon_{1}}^{+}}=(\alpha_{0}\beta_{0}\gamma_{0}|00010000\rangle +\alpha_{0}\beta_{0}\gamma_{1}|00011110\rangle \\ +\alpha_{0}\beta_{1}\gamma_{0}|00100000\rangle +\alpha_{0}\beta_{1}\gamma_{1}|00101110\rangle \;\\ +\alpha_{1}\beta_{0}\gamma_{0}|11010001\rangle +\alpha_{1}\beta_{0}\gamma_{1}|11011111\rangle \\ +\alpha_{1}\beta_{1}\gamma_{0}|11100001\rangle +\alpha_{1}\beta_{1}\gamma_{1}|11101111\rangle {)}_{B_{1}B_{2}C_{1}C_{2}A_{1}A_{2}A_{3}D} \end{array}$$

***Step 5.*** **The detailed processing for controller David**

After performing two three-qubit GHZ state measurements and a four-qubit GHZ state measurement, Alice, Bob, and Charlie communicate the outcomes of their results to the controller, David. Lastly, in order to ensure the smooth implementation of our scheme, David needs to perform a single-qubit von Neumann Z-base measurement on his qubit *D:*

(17) $$\left|\hbar^{+}\rangle_{D}=\frac{1}{\sqrt{2}}(|0\rangle +|1\rangle )\;|\hbar^{-}\rangle_{D}=\frac{1}{\sqrt{2}}(|0\rangle -|1\rangle \right)$$

If David’s outcome is |*ℏ^+^*⟩*D*, finally the state of the other particles (*B*_1_, *B*_2_, *C*_1_, *C*_2_, *A*_1_, *A*_2_, *A*_3_) will be collapsed into:

(18) $$\begin{array}{l} (\alpha_{0}\beta_{0}\gamma_{0}|0001000\rangle +\alpha_{0}\beta_{0}\gamma_{1}|0001111\rangle +\alpha_{0}\beta_{1}\gamma_{0}|0010000\rangle \\ +\alpha_{0}\beta_{1}\gamma_{1}|0010111\rangle +\alpha_{1}\beta_{0}\gamma_{0}|1101000\rangle +\alpha_{1}\beta_{0}\gamma_{1}|1101111\rangle \\ +\alpha_{1}\beta_{1}\gamma_{0}|1110000\rangle +\alpha_{1}\beta_{1}\gamma_{1}|1110111\rangle_{B_{1}B_{2}C_{1}C_{2}A_{1}A_{2}A_{3}})\\ ={\left(\alpha_{0}|00\rangle +\alpha_{1}|11\rangle \right)}_{B_{1}B_{2}}⊗{\left(\beta_{0}|01\rangle +\beta_{1}|10\rangle \right)}_{C_{1}C_{2}}⊗{\left(\gamma_{0}|000\rangle +\gamma_{1}|111\rangle \right)}_{A_{1}A_{2}A_{3}} \end{array}$$

***Step 6.*** **The corresponding scheme for recovering**

Lastly, the corresponding unitary transformation is executed. According to the final collapse state, if Alice, Bob, and Charlie undertake local unitary operations *I_B1_*⊗*I_B2_*⊗*I_C1_*⊗*I_C1_*⊗*I_A1_*⊗*I_A2_*⊗*I_A3_*, the desired state can be reconstructed. The successful completion of the ACCQT has been achieved.

There are a total of 128 possible cases with the same probability after all measurement operations in this scheme. Thus, the total success probability of the proposed scheme is 100%. In order to ensure the structure of the main text, all measurement states and corresponding unitary operations are listed in Appendix A, Table A1.

## 3. Fidelity Calculation

The fidelity is a metric based on the distance between two quantum states, indicating their level of closeness. As quantum states pass through environments with noise, some information is lost. This loss can be indirectly evaluated by determining the fidelity. In this particular section, we examine the impact of noise on the ACCQT process. We mathematically describe four types of noisy environments, including bit-flip, phase-flip, bit-phase-flip, and depolarizing environments that affect the quantum channel. We then analyze the quality of the protocol proposed by calculating the fidelities of the teleported states.

Considering that the controller David is only responsible for particle distribution. As a result, David’s particle does not have to transmit through the noisy channel, so it is not influenced by noise. To describe different sorts of noisy channels, the quantum channel is represented by a density operator using the Kraus operator [34].

(19) $$\rho =\sum E_{i}|\varphi \rangle_{B_{1}B_{2}B_{3}C_{1}C_{2}C_{3}A_{1}A_{2}A_{3}A_{4}D}\langle \varphi |E_{i}^{†}$$

The output state of the protocol is represented as:

(20) $$\rho^{\mathrm{out}}=U\left\{Tr_{B_{1}B_{2}C_{1}C_{2}A_{1}A_{2}A_{3}}\left[\left(\xi a_{1}a_{2}A_{4}⊗\xi b_{1}b_{2}B_{3}⊗\xi e_{1}e_{2}e_{3}C_{3}⊗\lambda_{D}\right)\rho_{B_{1}B_{2}B_{3}C_{1}C_{2}C_{3}A_{1}A_{2}A_{3}A_{4}D}⊗\rho a_{1}a_{2}⊗\rho b_{1}b_{2}⊗\rho e_{1}e_{2}e_{3}\right]\right\}U^{†}$$

In the above formula, U denotes unitary operations that are performed on the collapsed state after the measurements have been made. *ξa*_1_*a*_2_*A*_4_, *ξb*_1_*b*_2_*B*_3_, and *ξe*_1_*e*_2_*e*_3_*C*_3_ each represent a GHZ-state measurement for Alice, Bob, and Charlie. *λ_D_* represents the single-particle von Neumann measurement made by David.

Fidelity can be used to measure the accuracy and precision of quantum operations, as well as to assess the impact of noise channels on quantum teleportation. The fidelity value approaching one indicates minimal information loss, whereas a fidelity value closer to zero signifies a higher susceptibility to noise and greater information loss during transmission. The following is the formula for calculating fidelity:

(21) $$f=\langle \Theta \left|\rho^{\mathrm{out}}\right|\Theta \rangle$$

Here, |Θ⟩ represents the ideal quantum output state in a noiseless environment.

### 3.1. Bit-Flip Noisy Channel

The bit-flip noise channel is a category of noise that may arise during communication. In the course of transmitting quantum qubits through a bit-flip noisy channel, the state of the transmitted qubits might be altered by means of environmental interference or noise, resulting in bit flips. Consequently, the sent quantum bits no longer conform to the anticipated entangled state, thus negatively impacting the efficacy of quantum teleportation. In the above-mentioned noise channel, there exists a probability *p* (0 ≤ *p* ≤ 1) for quantum bits to incur bit-flip errors during transmission, while the probability of no error is 1 − *p*. The bit-flip noise can be represented using the Kraus operator provided in the following formula:

(22) $$E_{0}=\sqrt{1-p}\left(\begin{array}{cc} 1 & 0\\ 0 & 1 \end{array}\right)=\sqrt{1-p}I,E_{1}=\sqrt{p}\left(\begin{array}{cc} 0 & 1\\ 1 & 0 \end{array}\right)=\sqrt{p}X$$

After evolving in bit-flip noise, then we can determine the corresponding fidelity expression as:

(23) $$f=\langle \Theta \left|\rho^{out}\right|\Theta \rangle =\left[{\left(1-p\right)}^{10}+p^{10}\right]$$

**Figure 3:**
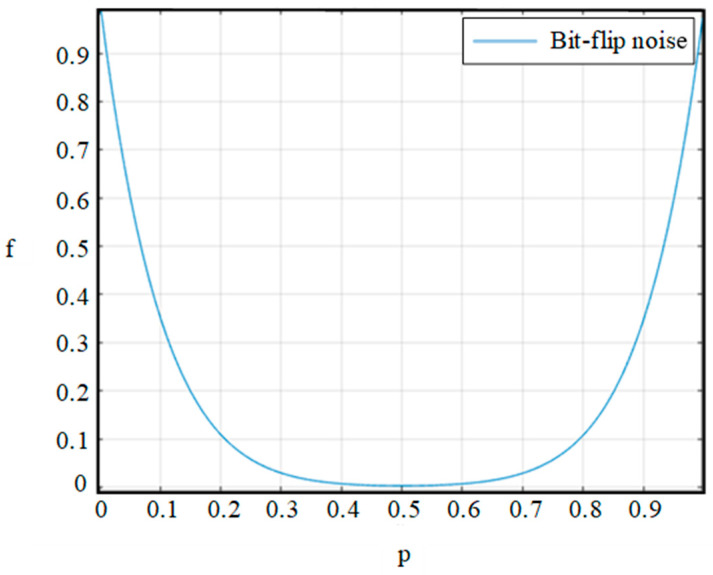
Effect of bit-flip noise on the ACCQT scheme is visualized through variation in fidelity f. In this figure, p means the success probability.

Based on the fidelity formula described above, we conclude that the fidelity of the protocol in the bit-flip noise channel is only dependent on the noise intensity. Additionally, by running the expression via MATLAB, as depicted in Figure 3, it can be visually observed that as the level of bit-flip noise increases, the fidelity exhibits a parabolic trend, initially decreasing and then increasing.

### 3.2. Phase-Flip Noisy Channel

The phase-flip noise channel is a common noisy scenario in quantum communication where the transmitted quantum qubits are prone to interference and errors. These errors result in each quantum qubit being flipped with a probability of *p*, meaning that |0⟩ becomes |1⟩ and |1⟩ becomes |0⟩. For the phase-flip noise, the Kraus operators are given by:

(24) $$E_{0}=\sqrt{1-p}\left(\begin{array}{cc} 1 & 0\\ 0 & 1 \end{array}\right)=\sqrt{1-p}I,E_{1}=\sqrt{p}\left(\begin{array}{cc} 1 & 0\\ 0 & -1 \end{array}\right)=\sqrt{p}Z$$

After the evolution in phase-flip noise, our final output state is given by:

(25) $$\begin{array}{l} \rho^{\mathrm{out}}={\left(1-p\right)}^{10}[\alpha_{0}\beta_{0}\gamma_{0}|0001000\rangle +\alpha_{0}\beta_{0}\gamma_{1}|0001111\rangle +\alpha_{0}\beta_{1}\gamma_{0}|0010000\rangle \\ +\alpha_{0}\beta_{1}\gamma_{1}|0010111\rangle +\alpha_{1}\beta_{0}\gamma_{0}|1101000\rangle +\alpha_{1}\beta_{0}\gamma_{1}|1101111\rangle +\alpha_{1}\beta_{1}\gamma_{0}|1110000\rangle \\ +\alpha_{1}\beta_{1}\gamma_{1}|1110111\rangle ]\;[\alpha_{0}\beta_{0}\gamma_{0}\langle 0001000|+\alpha_{0}\beta_{0}\gamma_{1}\langle 0001111|+\alpha_{0}\beta_{1}\gamma_{0}\langle 0010000|\\ +\alpha_{0}\beta_{1}\gamma_{1}\langle 0010111|+\alpha_{1}\beta_{0}\gamma_{0}\langle 1101000|+\alpha_{1}\beta_{0}\gamma_{1}\langle 1101111|+\alpha_{1}\beta_{1}\gamma_{0}\langle 1110000|\\ +\alpha_{1}\beta_{1}\gamma_{1}\langle 1110111|]+p^{10}[-\alpha_{0}\beta_{0}\gamma_{0}|0001000\rangle -\alpha_{0}\beta_{0}\gamma_{1}|0001111\rangle +\alpha_{0}\beta_{1}\gamma_{0}|0010000\rangle \\ +\alpha_{0}\beta_{1}\gamma_{1}|0010111\rangle +\alpha_{1}\beta_{0}\gamma_{0}|1101000\rangle +\alpha_{1}\beta_{0}\gamma_{1}|1101111\rangle -\alpha_{1}\beta_{1}\gamma_{0}|1110000\rangle \\ -\alpha_{1}\beta_{1}\gamma_{1}|1110111\rangle ][-\alpha_{0}\beta_{0}\gamma_{0}\langle 0001000|-\alpha_{0}\beta_{0}\gamma_{1}\langle 0001111|+\alpha_{0}\beta_{1}\gamma_{0}\langle 0010000|\\ +\alpha_{0}\beta_{1}\gamma_{1}\langle 0010111|+\alpha_{1}\beta_{0}\gamma_{0}\langle 1101000|+\alpha_{1}\beta_{0}\gamma_{1}\langle 1101111|-\alpha_{1}\beta_{1}\gamma_{0}\langle 1110000|\\ -\alpha_{1}\beta_{1}\gamma_{1}\langle 1110111|] \end{array}$$

Then, we can determine the corresponding fidelity expression as:

(26) $$f=\langle \Theta \left|\rho^{out}\right|\Theta \rangle =\left[{\left(1-p\right)}^{10}+p^{10}{\left(-\alpha_{0}^{2}\beta_{0}^{2}+\alpha_{0}^{2}\beta_{1}^{2}+\alpha_{1}^{2}\beta_{0}^{2}-\alpha_{1}^{2}\beta_{1}^{2}\right)}^{2}\right]$$

**Figure 4:**
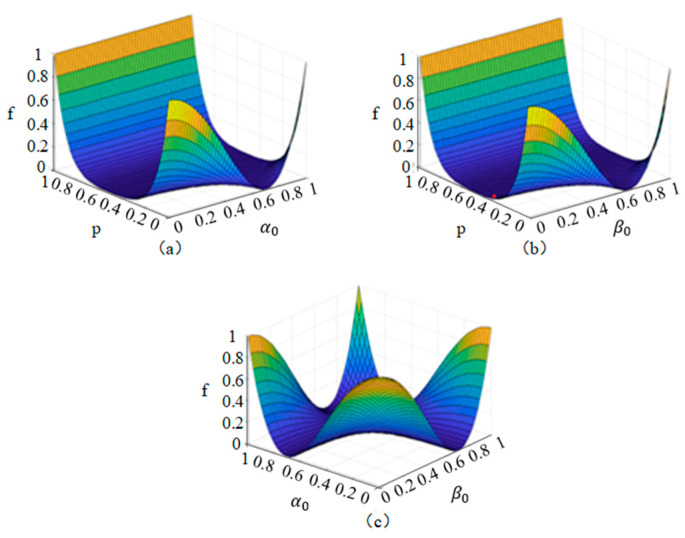
Analysis plot of fidelity f in a phase-flip noisy channel. In this figure, *p* is the success probability, and the parameters a and b represent the amplitude parameters of the transmitted quantum state.

Based on the above formula, we conclude that the fidelity of our protocol in phase-flip depends on two aspects: the noise intensity and amplitude parameters of the initial state. The fidelity of the protocol is shown in Figure 4. Here, *p* represents the noise intensity in the phase-flip noise channel, while *α*_0_, *α*_1_, *β*_0_, and *β*_1_ represent the amplitude parameters of the transmitted quantum state.

In Figure 4a, take *β*_0_ = 0, *β*_1_ = 1, and *α*_0_ = a; fidelity is only associated with the noise intensity *p* and amplitude parameter a of the initial state. When the noise intensity *p* is in the interval [0, 0.4], the fidelity decreases rapidly as the noise intensity increases, while the change in parameter a has almost no effect on the fidelity. The fidelity reaches the worst case when the noise intensity is around 0.6. When the noise intensity is in the interval [0.6, 1], the fidelity of the protocol decreases with parameter an up to a = 0.5, then the fidelity increases as an increase, reaching almost 1, indicating excellent communication transmission.

In Figure 4c, take the phase-flip noise intensity *p* = 1, *α*_0_, *β*_0_. At this time, the fidelity is related to parameters a and b. As can be seen from the figure, the image of fidelity change is a “concave” shape. As a and b increase, the fidelity will decrease first and then increase. The fidelity reaches a minimum when a and b are all around 0.6. It is worth noting that when one side of the parameters a and b tends to 1, and the other side tends to 0, a similar high fidelity can be achieved and complete quantum communication.

### 3.3. Bit-Phase-Flip Noisy Channel

The bit-phase-flip noise channel is a type of noise scenario that includes both bit-flip and phase-flip noises. In the bit-phase-flip noise channel, each qubit can undergo both bit-flip and phase-flip operations. Compared to other noise channels, the bit-phase-flip noise is more complex and can result in higher error rates. In the above-mentioned noise channel, there exists a probability *p* (0 ≤ *p* ≤ 1) for quantum bits to incur bit-phase-flip errors during transmission, while the probability of no error is 1 − *p*. The bit-phase-flip noise can be represented using the Kraus operator provided in the following formula:

(27) $$E_{0}=\sqrt{1-p}I\;E_{1}=\sqrt{p}Y=\sqrt{p}\left[\begin{array}{cc} 0 & -i\\ i & 0 \end{array}\right]$$

After the channel is affected by bit-phase-flip noise, subsequent measurement and unitary operations will continue, and the final output state can be expressed as:

(28) $$\begin{array}{l} \rho^{\mathrm{out}}={\left(1-p\right)}^{10}[\alpha_{0}\beta_{0}\gamma_{0}|0001000\rangle +\alpha_{0}\beta_{0}\gamma_{1}|0001111\rangle +\alpha_{0}\beta_{1}\gamma_{0}|0010000\rangle +\alpha_{0}\beta_{1}\gamma_{1}|0010111\rangle \\ +\alpha_{1}\beta_{0}\gamma_{0}|1101000\rangle +\alpha_{1}\beta_{0}\gamma_{1}|1101111\rangle +\alpha_{1}\beta_{1}\gamma_{0}|1110000\rangle +\alpha_{1}\beta_{1}\gamma_{1}|1110111\rangle ]\;\\ \left[\alpha_{0}\beta_{0}\gamma_{0}\langle 0001000|+\alpha_{0}\beta_{0}\gamma_{1}\langle 0001111|+\alpha_{0}\beta_{1}\gamma_{0}\langle 0010000|+\alpha_{0}\beta_{1}\gamma_{1}\langle 0010111\right|\\ +\alpha_{1}\beta_{0}\gamma_{0}\langle 1101000|+\alpha_{1}\beta_{0}\gamma_{1}\langle 1101111|+\alpha_{1}\beta_{1}\gamma_{0}\langle 1110000|+\alpha_{1}\beta_{1}\gamma_{1}\langle 1110111|]\\ +p^{10}[\alpha_{1}\beta_{1}\gamma_{1}|1110111\rangle +\alpha_{1}\beta_{1}\gamma_{0}|1110000\rangle -\alpha_{1}\beta_{0}\gamma_{1}|1101111\rangle -\alpha_{1}\beta_{0}\gamma_{0}|1101000\rangle \\ -\alpha_{0}\beta_{1}\gamma_{1}|0010111\rangle -\alpha_{0}\beta_{1}\gamma_{0}|0010000\rangle +\alpha_{0}\beta_{0}\gamma_{1}|0001111\rangle +\alpha_{0}\beta_{0}\gamma_{0}|0001000\rangle ]\\ \left[\alpha_{1}\beta_{1}\gamma_{1}\langle 1110111|+\alpha_{1}\beta_{1}\gamma_{0}\langle 1110000|-\alpha_{1}\beta_{0}\gamma_{1}\langle 1101111|-\alpha_{1}\beta_{0}\gamma_{0}\langle 1101000\right|\\ -\alpha_{0}\beta_{1}\gamma_{1}\langle 0010111|-\alpha_{0}\beta_{1}\gamma_{0}\langle 0010000|+\alpha_{0}\beta_{0}\gamma_{1}\langle 0001111|+\alpha_{0}\beta_{0}\gamma_{0}\langle 0001000|] \end{array}$$

Then, we can determine the corresponding fidelity expression as:

(29) $$f=\langle \Theta \left|\rho^{out}\right|\Theta \rangle =\left[{\left(1-p\right)}^{10}+p^{10}{\left(\alpha_{0}^{2}\beta_{0}^{2}-\alpha_{0}^{2}\beta_{1}^{2}-\alpha_{1}^{2}\beta_{0}^{2}+\alpha_{1}^{2}\beta_{1}^{2}\right)}^{2}\right]$$

**Figure 5:**
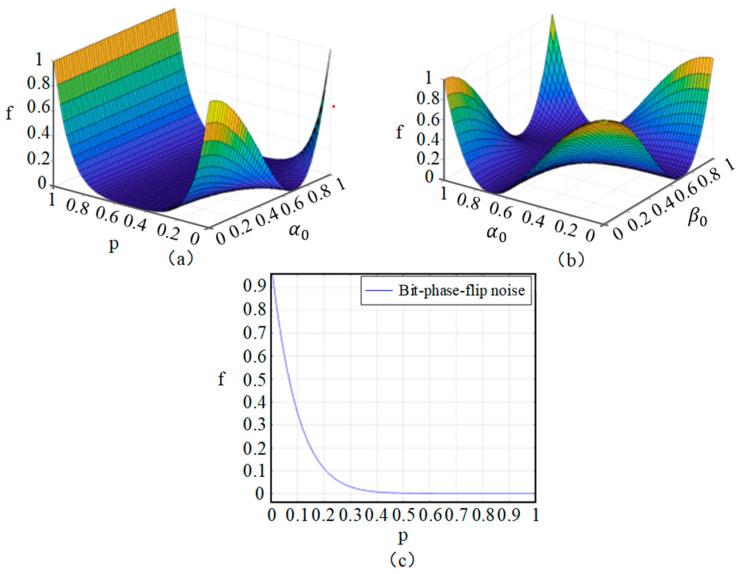
Analysis plot of fidelity f in a bit-phase-flip noisy channel. In this figure, p means the success probability, and the parameters a and b represent the amplitude parameters of the transmitted quantum state.

In the bit-phase-flip noise channel, the fidelity of the protocol is correlated with the noise intensity and the probability amplitude of the quantum state to be transmitted. As shown in Figure 5, it can visually reflect the variation in fidelity in the bit-phase-flip noise channel.

In Figure 5a, take *β*_0_ = 1, *β*_1_ = 0, and *α*_1_ = a. At this time, the fidelity is only associated with the noise intensity *p* and amplitude parameter a of the initial state. When the bit-phase-flip noise intensity *p* is within the range [0, 0.4], the variation in a has almost no effect on the fidelity, while the fidelity decreases rapidly with the increase in noise intensity. When the noise intensity *p* is around 0.5, the fidelity reaches the worst case, and quantum communication has long been out of reach.

In Figure 5b, the noise intensity of the bit-phase-flip noise is kept constant at 1, and *α*_0_ = a and *β*_0_ = b. At this point, fidelity is only related to a and b. From the figure, it can be seen that the fidelity change shows a “valley” shape, with high values around the edges and low values in the middle. As parameters a and b increase, the fidelity will decrease first and then increase. The fidelity performs well in all four “corners” of the figure, indicating that the quantum information transmitted through communication can be well-preserved in its original state, and the receiver can accurately read and decode this information.

In Figure 5c, when *α*_0_ = *β*_0_ = *α*_1_ = *β*_1_ and substituted into Equation (36), the fidelity of the protocol is only related to the intensity of the bit-phase-flip noise. When the channel noise intensity is in the interval [0, 0.2], the fidelity decreases sharply with the increase in the channel noise intensity. When the channel noise intensity is in the interval [0.4, 1], the fidelity tends to 0. Obviously, it can be found that the bit-phase-flip noise has a more drastic impact on its quantum fidelity than other types of noise.

### 3.4. Depolarizing Noisy Channel

In a depolarizing noisy channel, the qubits are depolarized with probability *p* (0 ≤ *p* ≤ 1), the system is left invariant with the probability 1 − *p*, while the operators X, Y, and Z act on the system with the probability $\frac{P}{3}$. The Kraus operators of depolarizing noise are as follows:

(30) $$E_{0}=\sqrt{1-\frac{3}{4}p}I,\;E_{1}=\frac{\sqrt{p}}{2}X,\;E_{2}=\frac{\sqrt{p}}{2}Y,\;E_{3}=\frac{\sqrt{p}}{2}Z$$

According to the Kraus operator representation of the depolarizing noise, after the channel is affected by depolarization noise, subsequent measurement, and unitary operations will continue; the expression for fidelity under the depolarizing noise channel can be determined as:

(31) $$f=\langle \Theta \left|\rho^{out}\right|\Theta \rangle ={\left(1-\frac{3}{4}p\right)}^{10}+\frac{p^{10}}{2^{20}}+2\times \frac{p^{10}}{2^{20}}{\left(\alpha_{0}^{2}\beta_{0}^{2}-\alpha_{0}^{2}\beta_{1}^{2}-\alpha_{1}^{2}\beta_{0}^{2}+\alpha_{1}^{2}\beta_{1}^{2}\right)}^{2}$$

Due to the negligible values of the last two items in Equation (31), the fidelity is simplified as:

(32) $$f=\langle \Theta \left|\rho^{out}\right|\Theta \rangle ={\left(1-\frac{3}{4}p\right)}^{10}$$

**Figure 6:**
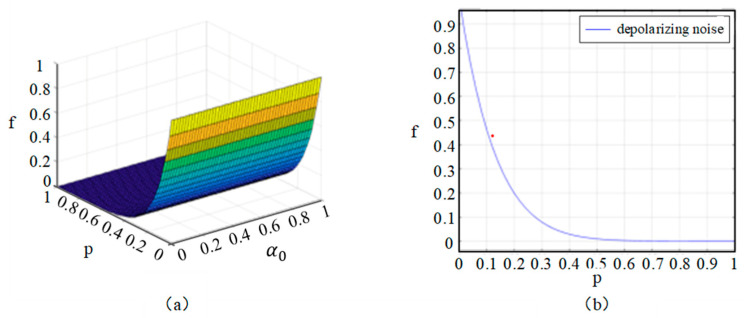
Analysis plot of fidelity f in a bit-phase-flip noisy channel. *p* is the success probability, and a represents the amplitude parameters of the transmitted quantum state.

In the depolarizing noise channel, qubits transmitted through it are prone to polarization transformations, leading the qubits to be randomly rotated to a new polarization direction. This new polarization direction may deviate from the original one, causing the receiver to be unable to accurately read and decode the qubit, ultimately resulting in transmission distortion or errors. Based on the fidelity measurements described above, we utilized MATLAB 2021a software to generate Figure 6, conducting a detailed noise analysis of our protocol in the depolarizing noise environment to ensure the stability of communication.

In Figure 6a, take *β*_0_ = 0, *β*_1_ = 1, and *α*_0_ = a. At this point, the fidelity is related to the channel noise intensity *p* and a. The fidelity decreases significantly with the noise intensity in the channel. It is worth noting that the parameter a has very little effect on the change in fidelity.

In Figure 6b, take *α*_0_ = *α*_1_ = *β*_0_ = *β*_1_. The fidelity is solely related to the channel noise intensity *p*. Analysis reveals that as the channel noise intensity *p* increases, the fidelity begins to slowly decrease. When *p* is in the interval [0, 0.05], the value of fidelity is still in a relatively ideal state. However, as *p* continues to increase, if the fidelity falls below 2/3, quantum teleportation cannot be achieved, resulting in communication transmission failure.

### 3.5. Comparison

**Figure 7:**
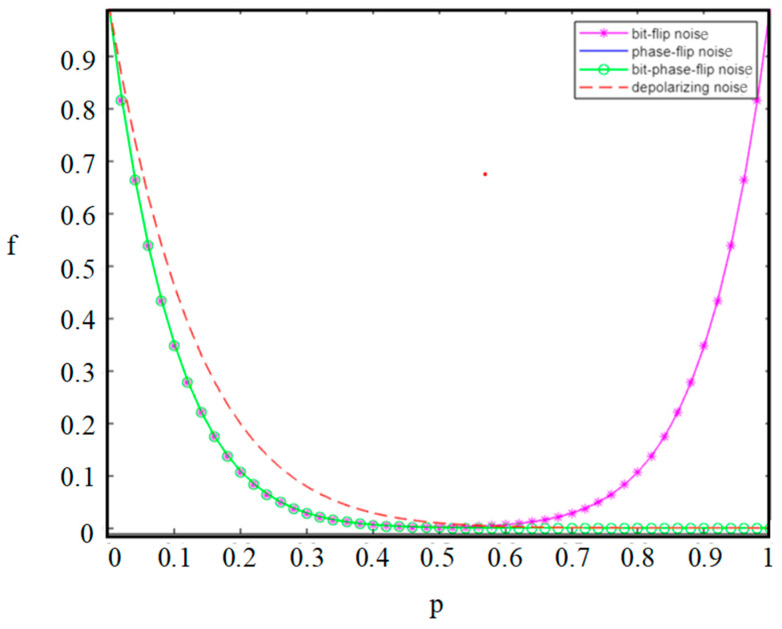
Analysis plot of fidelity f in four types of noisy channels, where *p* means the success probability.

In quantum communication, maintaining the purity and coherence of qubits is crucial for achieving secure long-distance communication and constructing efficient quantum networks in the future. To this end, we will compare and analyze our protocol under four different noise environments to determine the stable quantum information transmission of the protocol. In order to compare the trend of the fidelity of the output state with the change in the decoherence rate in four types of noisy environments. A complex two-dimensional graph of different curve colors is drawn in Figure 7.

Since the sensitivity of quantum systems to the environment, when particles are allocated to remote communication parties, the allocation process of quantum channels will inevitably be affected by noise, causing them to transition from pure states to mixed states. As a result, the coherence of the quantum channel decreases, particle information is lost, and fidelity is reduced. As the noise intensity p gradually increases, the quantum channel will turn into different quantum channels according to different noise scenarios. Here, we assume that noise intensity *p* is equal in four noise channels, and *α*_0_ = *α*_1_ = *β*_0_ = *β*_1_ =$\;\frac{\sqrt{2}}{2}$. The analysis reveals that in both phase-flip and bit-phase-flip noise environments, the curves overlap precisely, and their fidelity shows a consistent trend with the variation in noise intensity. In the case of the depolarization noise, the fidelity of the output state decreases as the decoherence rate increases. The fidelity decreases extremely rapidly at a noise intensity p greater than 0.1, at which point communication is interrupted. In addition, in the bit-flip noise environment, the fidelity exhibits a bimodal trend with increasing noise intensity (first decreasing and then increasing). When the noise intensity reaches 1, the quantum channel under bit-flip noise regains its coherence, which is contrary to intuition. Quantum coherence does not continue to decrease due to the increase in noise intensity. Instead, it first decreases and then increases. The reason is due to the effect of flipping, which is a difference compared to other noise environments. Moreover, according to the figure drawn by MATLAB, we find that compared with other noise environments, less information is lost when quantum communication is in the depolarizing noise environment. Note that the above analysis can be applied to real physical scenarios, and a perfect ACCQT can be achieved by assigning specific values of the initial state amplitude parameters.

## 4. Discussion and Analysis

### 4.1. Intrinsic Efficiency and Discussion

The intrinsic efficiency of our protocol is calculated as follows:

(33) $$\eta =\frac{q_{t}}{q_{c}+b_{c}}$$

where *q_t_* represents the total number of qubits that Alice, Bob, and Charlie want to teleport, *q_c_* indicates the number of qubits of the quantum channel, and *b_c_* represents the number of classical bits transmitted. *η* represents the intrinsic efficiency.

In Table 1, we compare the proposed scheme with the previous quantum teleportation protocol from seven aspects that are protocol type, quantum number used by quantum channel (QN), classical resource consumption (CRC), number of quantum bits transmitted (QBT), intrinsic efficiency (*η*), noise analysis (NA), and security analysis (SA).

CQT indicates controlled quantum teleportation, BCQT represents bidirectional controlled quantum teleportation, CCQT represents cyclic controlled quantum teleportation, and ABCQT represents asymmetric bidirectional controlled quantum teleportation. QT&RSP indicates a hybrid scheme of quantum teleportation and remote state preparation, and the ACCQT protocol proposed in this paper represents asymmetric cyclic controlled quantum teleportation.

From the above table, we see that our proposed protocol is quite different from other protocols. Firstly, our protocol can cyclically and asymmetrically transmit three completely different multi-qubit states simultaneously; it greatly improves the information capacity of communication and has better flexibility. Secondly, the present protocol is closer to the possibility of real-world experiments and is analyzed in a noisy environment. Furthermore, our proposed protocol has better intrinsic efficiency than other protocols. Finally, our protocol conducted security verification against two types of external attacks that quantum channels are susceptible to.

### 4.2. Security Analysis

In this section, we perform the security analysis of the provided protocol. Classical channels are, by default, encrypted with this system. Additionally, the quantum state cannot be transmitted across the quantum channel, making it impossible for Eve to eavesdrop on the transmission of quantum information. Thus, the quantum channel attack only occurred *during* entanglement distribution, where Alice holds four qubits (A_1_, A_2_, A_3_, A_4_), and Bob and Charlie each hold three qubits (B_1_, B_2_, B_3_) and (C_1_, C_2_, C_3_) under the allocation of the controller David. Regarding the two most frequent quantum channel attacks (the eavesdropping attack and the intercept-resend attack), we discuss them, respectively, as follows.

At first, we assume that the quantum channel suffers from eavesdropping attacks. Eavesdropper entangles his three auxiliary particles, V_1_, V_2,_ and V_3,_ with David’s qubit D, where the initial states of V_1_, V_2,_ and V_3_ are $|0\rangle_{V_{1}}$, $|0\rangle_{V_{2}}$ and $|0\rangle_{V_{3}}$. Then, the eavesdropper applies measurements on his three auxiliary particles to acquire secret information. Under the condition that Alice, Bob, Charlie, and David are all oblivious of the existence of the eavesdropper, assume the measurement outcomes of Alice, Bob, and Charlie are |*δ*^+^⟩*a_0_a_1_A_4_*, |*ϖ*^+^⟩*b_1_b_2_B_3_*, |*ε*^+^⟩*c_1_c_2_c_3_C_3_*, respectively. Then, the entire quantum system will collapse into:

(34) $$\begin{array}{l} \langle \delta^{+}{|}_{a_{1}a_{2}A_{4}}\langle \omega^{+}{|}_{b_{1}b_{2}B_{3}}{\langle \varepsilon^{+}{|}_{c_{1}c_{2}c_{3}C_{3}}\Psi \rangle }_{B_{1}B_{2}B_{3}C_{1}C_{2}C_{3}A_{1}A_{2}A_{3}A_{4}DV_{1}V_{2}V_{3}}\\ =\left(\alpha_{0}\beta_{0}\gamma_{0}|00010000000\rangle +\alpha_{0}\beta_{0}\gamma_{1}|0001110000\rangle +\alpha_{0}\beta_{1}\gamma_{0}|0010000000\rangle \right.\\ +\alpha_{0}\beta_{1}\gamma_{1}|00101110000\rangle +\alpha_{1}\beta_{0}\gamma_{0}|11010001000\rangle +\alpha_{1}\beta_{0}\gamma_{1}|11011111000\rangle \\ +\left.\alpha_{1}\beta_{1}\gamma_{0}|11100001000\rangle +\alpha_{1}\beta_{1}\gamma_{1}|11101111000\rangle_{B_{1}B_{2}C_{1}C_{2}A_{1}A_{2}A_{3}A_{3}DV_{1}V_{2}V_{3}}\right) \end{array}$$

If David’s measurement result is |*ℏ*^+^⟩*_D_*, the total state will change into:

(35) $$\begin{array}{l} \langle \hbar^{+}{|}_{D}\langle \delta^{+}{|}_{a_{1}a_{2}A_{4}}\langle \omega^{+}{|}_{b_{1}b_{2}B_{3}}{\langle \varepsilon^{+}{|}_{c_{1}c_{2}c_{3}C_{3}}\Psi \rangle }_{B_{1}B_{2}B_{3}C_{1}C_{2}C_{3}A_{1}A_{2}A_{3}A_{4}DV_{1}V_{2}V_{3}}\\ =\left(\alpha_{0}\beta_{0}\gamma_{0}|0001000000\rangle +\alpha_{0}\beta_{0}\gamma_{1}|000111000\rangle +\alpha_{0}\beta_{1}\gamma_{0}|001000000\rangle \right.\\ +\alpha_{0}\beta_{1}\gamma_{1}|0010111000\rangle +\alpha_{1}\beta_{0}\gamma_{0}|1101000000\rangle +\alpha_{1}\beta_{0}\gamma_{1}|1101111000\rangle \\ +\left.\alpha_{1}\beta_{1}\gamma_{0}|1110000000\rangle +\alpha_{1}\beta_{1}\gamma_{1}|1110111000\rangle_{B_{1}B_{2}C_{1}C_{2}A_{1}A_{2}A_{3}A_{3}DV_{1}V_{2}V_{3}}\right)\\ ={\left(\alpha_{0}|00\rangle +\alpha_{1}|11\rangle \right)}_{B_{1}B_{2}}⊗{\left(\beta_{0}|01\rangle +\beta_{1}|10\rangle \right)}_{C_{1}C_{2}}⊗{\left(\gamma_{0}|000\rangle +\gamma_{1}|111\rangle \right)}_{A_{1}A_{2}A_{3}}\\ ⊗|000\rangle_{V_{1}V_{2}V_{3}} \end{array}$$

while if David’s measurement result is |*ℏ*^−^⟩*_D_*, the total state will collapse into:

(36) $$\begin{array}{l} \langle \hbar^{-}{|}_{D}\langle \delta^{+}{|}_{a_{1}a_{2}A_{4}}\langle \omega^{+}{|}_{b_{1}b_{2}B_{3}}{\langle \varepsilon^{+}{|}_{c_{1}c_{2}c_{3}C_{3}}\Psi \rangle }_{B_{1}B_{2}B_{3}C_{1}C_{2}C_{3}A_{1}A_{2}A_{3}A_{4}DV_{1}V_{2}V_{3}}\\ =\left(\alpha_{0}\beta_{0}\gamma_{0}|0001000000\rangle +\alpha_{0}\beta_{0}\gamma_{1}|000111000\rangle +\alpha_{0}\beta_{1}\gamma_{0}|001000000\rangle \right.\\ +\alpha_{0}\beta_{1}\gamma_{1}|0010111000\rangle -\alpha_{1}\beta_{0}\gamma_{0}|1101000000\rangle -\alpha_{1}\beta_{0}\gamma_{1}|1101111000\rangle \\ -\left.\alpha_{1}\beta_{1}\gamma_{0}|1110000000\rangle -\alpha_{1}\beta_{1}\gamma_{1}|1110111000\rangle_{B_{1}B_{2}C_{1}C_{2}A_{1}A_{2}A_{3}A_{3}DV_{1}V_{2}V_{3}}\right)\\ ={\left(\alpha_{0}|00\rangle -\alpha_{1}|11\rangle \right)}_{B_{1}B_{2}}⊗{\left(\beta_{0}|01\rangle +\beta_{1}|10\rangle \right)}_{C_{1}C_{2}}⊗{\left(\gamma_{0}|000\rangle +\gamma_{1}|111\rangle \right)}_{A_{1}A_{2}A_{3}}\\ ⊗|000\rangle_{V_{1}V_{2}V_{3}} \end{array}$$

According to the above two formulas, whatever David’s measurement outcome is, the states of V_1_, V_2,_ and V_3_ are not entangled with Alice, Bob, and Charlie. Thus, eavesdropping cannot intercept any quantum information from three observers.

Then, we discuss intercept resend attacks. Before entanglement distribution, David prepares some single-qubit decoy states, which are generated randomly from the basis {|0⟩,$\left|1\right\rangle$,$\;\left|+\right\rangle$,$\left|-\right\rangle$}, where $\left|+\right\rangle \;$and $\left|-\right\rangle$ are measured on the X-basis, and $\left|0\right\rangle \;$and $\left|1\right\rangle \;$are measured on the Z-basis. Assume Eve intercepts qubits (A_1_, A_2_, A_3_, A_4_) during entanglement distribution. Next, he prepares another entangled state of eleven qubits and transmits the qubits (A_1_, A_2_, A_3_, A_4_) to Alice. Afterward, David placed the single-qubit decoy states into the qubit sequence in a specific order (A_1_, A_2_, A_3_, A_4_), which was unknown to the eavesdropper. After Alice received the eavesdropped qubit sequence (A_1_, A_2_, A_3_, A_4_), she notified David, and then David announced the specific location and measurement basis of the decoy state to Alice through the classical channel. Alice then measures the bait status on the correct basis and sends the measurements back to David. Based on the measurement results, David can determine whether there is an eavesdropper stealing information and use this to decide whether to interrupt the communication. So, our proposed scheme can be guaranteed in terms of security.

## 5. Conclusions

In previous works, all of which only conducted research on symmetric information exchange. In their schemes, the number of quantum states transmitted is the same. This quantum communication model has some limitations when transmitting quantum states. In response to this, a novel ACCQT protocol is proposed in this paper, which can cyclically teleport three asymmetric unknown, different two two-qubit and one three-qubit states at the same time. The information capacity of communication is greatly increased, and each correspondent can send different numbers of quantum states, which can satisfy the requirement of transporting diverse quantum information and provide a more flexible transmission model in quantum network communication. To achieve this aim, an eleven-qubit entangled state is employed as the quantum channel shared among four participants: Alice, Bob, and Charlie, and a controller, David, in advance. Then, the proposed protocol is analyzed in a typical verification environment, and by comparing various noisy environments, it is found that, for a particular choice of transmitted state, the loss of information is less in a depolarizing noisy channel; the fidelities of the ACCQT protocol only depend on two factors: the amplitude parameter of the initial state and the decoherence rate.

In addition, based on the security check of BB84 quantum key distribution, we conduct security analysis. Due to the existence of the controller David and the nonclonability of quantum, the two mainstream quantum channel attacks, such as eavesdropping attacks and intercept-resend attacks, can be prevented, and the secure transmission of information can be realized. Furthermore, in terms of theory and intrinsic efficiency, comparing peer papers, we illustrate the feasibility and advantage of the scheme. It is hoped that this research can provide some help for further establishing efficient quantum communication networks in the future.

## Figures and Tables

**Table 1:** Comparison of proposed protocol with previous schemes.

Scheme	Type of Protocol	QN	CRC	QBT	*η*	SA	NA
[35]	CQT	3	3	1	16.6%	No	No
[36]	BCQT	7	7	3	21.4%	No	No
[37]	CCQT	7	9	3	18.6%	No	No
[38]	ABCQT	11	9	6	30.0%	No	No
[39]	ABCQT	12	12	6	25.0%	No	No
[40]	QT&RSP	15	12	6	22.0%	Yes	No
Our	ACCQT	11	10	7	33.3%	Yes	Yes

## Data Availability

No data was used in this study.

## Appendix A

**Table A1:** The measurement results of users and the specific unitary transformation.

Alice’s Result	Bob’s Result	Charlie’s Result	David’s Result	Unitary Operation
|*δ*^+^⟩a_1_a_2_A_4_	|*ω*^+^⟩*b_1_b_2_B_3_*	|*ε*^+^⟩*c_1_c_2_c_3_C_3_*	|*ℏ*^±^⟩*D*	*I*⊗*I*⊗*I*⊗*I*⊗*I*⊗*I*⊗*I* *Z*⊗*I*⊗*I*⊗*I*⊗*I*⊗*I*⊗*I*
|*δ*^+^⟩a_1_a_2_A_4_	|*ω*^+^⟩*b_1_b_2_B_3_*	|*ε*^−^⟩*c_1_c_2_c_3_C_3_*	|*ℏ*^±^⟩*D*	*I*⊗*I*⊗*I*⊗*I*⊗*Z*⊗*I*⊗*I* *Z*⊗*I*⊗*I*⊗*I*⊗*Z*⊗*I*⊗*I*
|*δ*^+^⟩a_1_a_2_A_4_	|*ω*^+^⟩*b_1_b_2_B_3_*	|*ℓ^+^*⟩*c_1_c_2_c_3_C_3_*	|*ℏ*^±^⟩*D*	*I*⊗*I*⊗*I*⊗*I*⊗*X*⊗*X*⊗*X* *Z*⊗*I*⊗*I*⊗*I*⊗*X*⊗*X*⊗*X*
|*δ*^+^⟩a_1_a_2_A_4_	|*ω*^+^⟩*b_1_b_2_B_3_*	|*ℓ^−^*⟩*c_1_c_2_c_3_C_3_*	|*ℏ*^±^⟩*D*	*I*⊗*I*⊗*I*⊗*I*⊗*-iY*⊗*X*⊗*X* *Z*⊗*I*⊗*I*⊗*I*⊗*-iY⊗X⊗X*
|*δ*^+^⟩a_1_a_2_A_4_	|*ω*^−^⟩*b_1_b_2_B_3_*	|*ε*^+^⟩*c_1_c_2_c_3_C_3_*	|*ℏ*^±^⟩*D*	*I*⊗*I*⊗*Z*⊗*I*⊗*I*⊗*I*⊗*I* *Z*⊗*I*⊗*Z*⊗*I*⊗*I*⊗*I*⊗*I*
|*δ*^+^⟩a_1_a_2_A_4_	|*ω*^−^⟩*b_1_b_2_B_3_*	|*ε*^−^⟩*c_1_c_2_c_3_C_3_*	|*ℏ*^±^⟩*D*	*I*⊗*I*⊗*Z*⊗*I*⊗*Z*⊗*I*⊗*I* *Z*⊗*I*⊗*Z*⊗*I*⊗*Z*⊗*I*⊗*I*
|*δ*^+^⟩a_1_a_2_A_4_	|*ω*^−^⟩*b_1_b_2_B_3_*	|*ℓ^+^*⟩*c_1_c_2_c_3_C_3_*	|*ℏ*^±^⟩*D*	*I*⊗*I*⊗*Z*⊗*I*⊗*X*⊗*X*⊗*X* *Z*⊗*I*⊗*Z*⊗*I*⊗*X*⊗*X*⊗*X*
|*δ*^+^⟩a_1_a_2_A_4_	|*ω*^−^⟩*b_1_b_2_B_3_*	|*ℓ^−^*⟩*c_1_c_2_c_3_C_3_*	|*ℏ*^±^⟩*D*	*I*⊗*I*⊗*Z*⊗*I*⊗*-iY*⊗*X*⊗*X* *Z*⊗*I*⊗*Z*⊗*I*⊗*-iY⊗X⊗X*
|*δ*^+^⟩a_1_a_2_A_4_	|*μ*^+^⟩*b_1_b_2_B_3_*	|*ε*^+^⟩*c_1_c_2_c_3_C_3_*	|*ℏ*^±^⟩*D*	*I*⊗*I*⊗*X*⊗*X*⊗*I*⊗*I*⊗*I* *Z*⊗*I*⊗*X*⊗*X*⊗*I*⊗*I*⊗*I*
|*δ*^+^⟩a_1_a_2_A_4_	|*μ*^+^⟩*b_1_b_2_B_3_*	|*ε*^−^⟩*c_1_c_2_c_3_C_3_*	|*ℏ*^±^⟩*D*	*I*⊗*I*⊗*X*⊗*X*⊗*Z*⊗*I*⊗*I* *Z*⊗*I*⊗*X*⊗*X*⊗*Z*⊗*I*⊗*I*
|*δ*^+^⟩a_1_a_2_A_4_	|*μ*^+^⟩*b_1_b_2_B_3_*	|*ℓ^+^*⟩*c_1_c_2_c_3_C_3_*	|*ℏ*^±^⟩*D*	*I*⊗*I*⊗*X*⊗*X*⊗*X*⊗*X*⊗*X* *Z*⊗*I*⊗*X*⊗*X*⊗*X*⊗*X*⊗*X*
|*δ*^+^⟩a_1_a_2_A_4_	|*μ*^+^⟩*b_1_b_2_B_3_*	|*ℓ^−^*⟩*c_1_c_2_c_3_C_3_*	|*ℏ*^±^⟩*D*	*I*⊗*I*⊗*X*⊗*X*⊗*-iY*⊗*X*⊗*X* *Z*⊗*I*⊗*X*⊗*X*⊗*-iY⊗X⊗X*
|*δ*^+^⟩a_1_a_2_A_4_	|*μ*^−^⟩*b_1_b_2_B_3_*	|*ε*^+^⟩*c_1_c_2_c_3_C_3_*	|*ℏ*^±^⟩*D*	*I*⊗*I*⊗*-iY*⊗*X*⊗*I*⊗*I*⊗*I* *Z*⊗*I*⊗*-iY*⊗*X*⊗*I*⊗*I*⊗*I*
|*δ*^+^⟩a_1_a_2_A_4_	|*μ*^−^⟩*b_1_b_2_B_3_*	|*ε*^−^⟩*c_1_c_2_c_3_C_3_*	|*ℏ*^±^⟩*D*	*I*⊗*I*⊗*-iY*⊗*X*⊗*Z*⊗*I*⊗*I* *Z*⊗*I*⊗*-iY*⊗*X*⊗*Z*⊗*I*⊗*I*
|*δ*^+^⟩a_1_a_2_A_4_	|*μ*^−^⟩*b_1_b_2_B_3_*	|*ℓ^+^*⟩*c_1_c_2_c_3_C_3_*	|*ℏ*^±^⟩*D*	*I*⊗*I*⊗*-iY⊗X*⊗*X*⊗*X*⊗*X* *Z*⊗*I*⊗*-iY*⊗*X*⊗*X*⊗*X*⊗*X*
|*δ*^+^⟩a_1_a_2_A_4_	|*μ*^−^⟩*b_1_b_2_B_3_*	|*ℓ^−^*⟩*c_1_c_2_c_3_C_3_*	|*ℏ*^±^⟩*D*	*I*⊗*I*⊗*-iY*⊗*X*⊗*-iY*⊗*X*⊗*X* *Z*⊗*I*⊗*-iY⊗X*⊗*-iY⊗X⊗X*
|*δ^−^*⟩a_1_a_2_A_4_	|*ω*^+^⟩*b_1_b_2_B_3_*	|*ε*^+^⟩*c_1_c_2_c_3_C_3_*	|*ℏ*^±^⟩*D*	*Z*⊗*I*⊗*I*⊗*I*⊗*I*⊗*I*⊗*I* *I*⊗*I*⊗*I*⊗*I*⊗*I*⊗*I*⊗*I*
|*δ^−^*⟩a_1_a_2_A_4_	|*ω*^+^⟩*b_1_b_2_B_3_*	|*ε*^−^⟩*c_1_c_2_c_3_C_3_*	|*ℏ*^±^⟩*D*	*Z*⊗*I*⊗*I*⊗*I*⊗*Z*⊗*I*⊗*I* *I*⊗*I*⊗*I*⊗*I*⊗*Z*⊗*I*⊗*I*
|*δ^−^*⟩a_1_a_2_A_4_	|*ω*^+^⟩*b_1_b_2_B_3_*	|*ℓ^+^*⟩*c_1_c_2_c_3_C_3_*	|*ℏ*^±^⟩*D*	*Z*⊗*I*⊗*I*⊗*I*⊗*X*⊗*X*⊗*X* *I*⊗*I*⊗*I*⊗*I*⊗*X*⊗*X*⊗*X*
|*δ^−^*⟩a_1_a_2_A_4_	|*ω*^+^⟩*b_1_b_2_B_3_*	|*ℓ^−^*⟩*c_1_c_2_c_3_C_3_*	|*ℏ*^±^⟩*D*	*Z*⊗*I*⊗*I*⊗*I*⊗*-iY*⊗*X*⊗*X* *I*⊗*I*⊗*I*⊗*I*⊗*-iY⊗X⊗X*
|*δ^−^*⟩a_1_a_2_A_4_	|*ω*^−^⟩*b_1_b_2_B_3_*	|*ε*^+^⟩*c_1_c_2_c_3_C_3_*	|*ℏ*^±^⟩*D*	*Z*⊗*I*⊗*Z*⊗*I*⊗*I*⊗*I*⊗*I* *I*⊗*I*⊗*Z*⊗*I*⊗*I*⊗*I*⊗*I*
|*δ^−^*⟩a_1_a_2_A_4_	|*ω*^−^⟩*b_1_b_2_B_3_*	|*ε*^−^⟩*c_1_c_2_c_3_C_3_*	|*ℏ*^±^⟩*D*	*Z*⊗*I*⊗*Z*⊗*I*⊗*Z*⊗*I*⊗*I* *I*⊗*I*⊗*Z*⊗*I*⊗*Z*⊗*I*⊗*I*
|*δ^−^*⟩a_1_a_2_A_4_	|*ω*^−^⟩*b_1_b_2_B_3_*	|*ℓ^+^*⟩*c_1_c_2_c_3_C_3_*	|*ℏ*^±^⟩*D*	*Z*⊗*I*⊗*Z*⊗*I*⊗*X*⊗*X*⊗*X* *I*⊗*I*⊗*Z*⊗*I*⊗*X*⊗*X*⊗*X*
|*δ^−^*⟩a_1_a_2_A_4_	|*ω*^−^⟩*b_1_b_2_B_3_*	|*ℓ^−^*⟩*c_1_c_2_c_3_C_3_*	|*ℏ*^±^⟩*D*	*Z*⊗*I*⊗*Z*⊗*I*⊗*-iY*⊗*X*⊗*X* *I*⊗*I*⊗*Z*⊗*I*⊗*-iY⊗X⊗X*
|*δ^−^*⟩a_1_a_2_A_4_	|*μ*^+^⟩*b_1_b_2_B_3_*	|*ε*^+^⟩*c_1_c_2_c_3_C_3_*	|*ℏ*^±^⟩*D*	*Z*⊗*I*⊗*X*⊗*X*⊗*I*⊗*I*⊗*I* *I*⊗*I*⊗*X*⊗*X*⊗*I*⊗*I*⊗*I*
|*δ^−^*⟩a_1_a_2_A_4_	|*μ*^+^⟩*b_1_b_2_B_3_*	|*ε*^−^⟩*c_1_c_2_c_3_C_3_*	|*ℏ*^±^⟩*D*	*Z*⊗*I*⊗*X*⊗*X*⊗*Z*⊗*I*⊗*I* *I*⊗*I*⊗*X*⊗*X*⊗*Z*⊗*I*⊗*I*
|*δ^−^*⟩a_1_a_2_A_4_	|*μ*^+^⟩*b_1_b_2_B_3_*	|*ℓ^+^*⟩*c_1_c_2_c_3_C_3_*	|*ℏ*^±^⟩*D*	*Z*⊗*I*⊗*X*⊗*X*⊗*X*⊗*X*⊗*X* *I*⊗*I*⊗*X*⊗*X*⊗*X*⊗*X*⊗*X*
|*δ^−^*⟩a_1_a_2_A_4_	|*μ*^+^⟩*b_1_b_2_B_3_*	|*ℓ^−^*⟩*c_1_c_2_c_3_C_3_*	|*ℏ*^±^⟩*D*	*Z*⊗*I*⊗*X*⊗*X*⊗*-iY*⊗*X*⊗*X* *I*⊗*I*⊗*X*⊗*X*⊗*-iY⊗X⊗X*
|*δ^−^*⟩a_1_a_2_A_4_	|*μ*^−^⟩*b_1_b_2_B_3_*	|*ε*^+^⟩*c_1_c_2_c_3_C_3_*	|*ℏ*^±^⟩*D*	*Z*⊗*I*⊗*-iY*⊗*X*⊗*I*⊗*I*⊗*I* *I*⊗*I*⊗*-iY*⊗*X*⊗*I*⊗*I*⊗*I*
|*δ^−^*⟩a_1_a_2_A_4_	|*μ*^−^⟩*b_1_b_2_B_3_*	|*ε*^−^⟩*c_1_c_2_c_3_C_3_*	|*ℏ*^±^⟩*D*	*Z*⊗*I*⊗*-iY*⊗*X*⊗*Z*⊗*I*⊗*I* *I*⊗*I*⊗*-iY*⊗*X*⊗*Z*⊗*I*⊗*I*
|*δ^−^*⟩a_1_a_2_A_4_	|*μ*^−^⟩*b_1_b_2_B_3_*	|*ℓ^+^*⟩*c_1_c_2_c_3_C_3_*	|*ℏ*^±^⟩*D*	*Z*⊗*I*⊗*-iY⊗X*⊗*X*⊗*X*⊗*X* *I*⊗*I*⊗*-iY*⊗*X*⊗*X*⊗*X*⊗*X*
|*δ^−^*⟩a_1_a_2_A_4_	|*μ*^−^⟩*b_1_b_2_B_3_*	|*ℓ^−^*⟩*c_1_c_2_c_3_C_3_*	|*ℏ*^±^⟩*D*	*Z*⊗*I*⊗*-iY*⊗*X*⊗*-iY*⊗*X*⊗*X* *I*⊗*I*⊗*-iY⊗X*⊗*-iY⊗X⊗X*
|*η*^+^⟩a_1_a_2_A_4_	|*ω*^+^⟩*b_1_b_2_B_3_*	|*ε*^+^⟩*c_1_c_2_c_3_C_3_*	|*ℏ*^±^⟩*D*	*X*⊗*X*⊗*I*⊗*I*⊗*I*⊗*I*⊗*I* *-iY*⊗*X*⊗*I*⊗*I*⊗*I*⊗*I*⊗*I*
|*η*^+^⟩a_1_a_2_A_4_	|*ω*^+^⟩*b_1_b_2_B_3_*	|*ε*^−^⟩*c_1_c_2_c_3_C_3_*	|*ℏ*^±^⟩*D*	*X*⊗*X*⊗*I*⊗*I*⊗*Z*⊗*I*⊗*I* *-iY*⊗*X*⊗*I*⊗*I*⊗*Z*⊗*I*⊗*I*
|*η*^+^⟩a_1_a_2_A_4_	|*ω*^+^⟩*b_1_b_2_B_3_*	|*ℓ^+^*⟩*c_1_c_2_c_3_C_3_*	|*ℏ*^±^⟩*D*	*X⊗X*⊗*I*⊗*I*⊗*X*⊗*X*⊗*X* *-iY⊗X*⊗*I*⊗*I*⊗*X*⊗*X*⊗*X*
|*η*^+^⟩a_1_a_2_A_4_	|*ω*^+^⟩*b_1_b_2_B_3_*	|*ℓ^−^*⟩*c_1_c_2_c_3_C_3_*	|*ℏ*^±^⟩*D*	*X*⊗*X*⊗*I*⊗*I*⊗*-iY*⊗*X*⊗*X* *-iY*⊗*X*⊗*I*⊗*I*⊗*-iY⊗X⊗X*
|*η*^+^⟩a_1_a_2_A_4_	|*ω*^−^⟩*b_1_b_2_B_3_*	|*ε*^+^⟩*c_1_c_2_c_3_C_3_*	|*ℏ*^±^⟩*D*	*X*⊗*X*⊗*Z*⊗*I*⊗*I*⊗*I*⊗*I* *-iY*⊗*X*⊗*Z*⊗*I*⊗*I*⊗*I*⊗*I*
|*η*^+^⟩a_1_a_2_A_4_	|*ω*^−^⟩*b_1_b_2_B_3_*	|*ε*^−^⟩*c_1_c_2_c_3_C_3_*	|*ℏ*^±^⟩*D*	*X*⊗*X*⊗*Z*⊗*I*⊗*Z*⊗*I*⊗*I* *-iY*⊗*X*⊗*Z*⊗*I*⊗*Z*⊗*I*⊗*I*
|*η*^+^⟩a_1_a_2_A_4_	|*ω*^−^⟩*b_1_b_2_B_3_*	|*ℓ^+^*⟩*c_1_c_2_c_3_C_3_*	|*ℏ*^±^⟩*D*	*X*⊗*X*⊗*Z*⊗*I*⊗*X*⊗*X*⊗*X* *-iY*⊗*X*⊗*Z*⊗*I*⊗*X*⊗*X*⊗*X*
|*η*^+^⟩a_1_a_2_A_4_	|*ω*^−^⟩*b_1_b_2_B_3_*	|*ℓ^−^*⟩*c_1_c_2_c_3_C_3_*	|*ℏ*^±^⟩*D*	*X*⊗*X*⊗*Z*⊗*I*⊗*-iY*⊗*X*⊗*X* *-iY*⊗*X*⊗*Z*⊗*I*⊗*-iY⊗X⊗X*
|*η*^+^⟩a_1_a_2_A_4_	|*μ*^+^⟩*b_1_b_2_B_3_*	|*ε*^+^⟩*c_1_c_2_c_3_C_3_*	|*ℏ*^±^⟩*D*	*X*⊗*X*⊗*X*⊗*X*⊗*I*⊗*I*⊗*I* *-iY*⊗*X*⊗*X*⊗*X*⊗*I*⊗*I*⊗*I*
|*η*^+^⟩a_1_a_2_A_4_	|*μ*^+^⟩*b_1_b_2_B_3_*	|*ε*^−^⟩*c_1_c_2_c_3_C_3_*	|*ℏ*^±^⟩*D*	*X*⊗*X*⊗*X*⊗*X*⊗*Z*⊗*I*⊗*I* *-iY*⊗*X*⊗*X*⊗*X*⊗*Z*⊗*I*⊗*I*
|*η*^+^⟩a_1_a_2_A_4_	|*μ*^+^⟩*b_1_b_2_B_3_*	|*ℓ^+^*⟩*c_1_c_2_c_3_C_3_*	|*ℏ*^±^⟩*D*	*X*⊗*X*⊗*X*⊗*X*⊗*X*⊗*X*⊗*X* *-iY*⊗*X*⊗*X*⊗*X*⊗*X*⊗*X*⊗*X*
|*η*^+^⟩a_1_a_2_A_4_	|*μ*^+^⟩*b_1_b_2_B_3_*	|*ℓ^−^*⟩*c_1_c_2_c_3_C_3_*	|*ℏ*^±^⟩*D*	*X*⊗*X*⊗*X*⊗*X*⊗*-iY*⊗*X*⊗*X* *-iY*⊗*X*⊗*X*⊗*X*⊗*-iY⊗X⊗X*
|*η*^+^⟩a_1_a_2_A_4_	|*μ*^−^⟩*b_1_b_2_B_3_*	|*ε*^+^⟩*c_1_c_2_c_3_C_3_*	|*ℏ*^±^⟩*D*	*X*⊗*X*⊗*-iY*⊗*X*⊗*I*⊗*I*⊗*I* *-iY*⊗*X*⊗*-iY*⊗*X*⊗*I*⊗*I*⊗*I*
|*η*^+^⟩a_1_a_2_A_4_	|*μ*^−^⟩*b_1_b_2_B_3_*	|*ε*^−^⟩*c_1_c_2_c_3_C_3_*	|*ℏ*^±^⟩*D*	*X*⊗*X*⊗*-iY*⊗*X*⊗*Z*⊗*I*⊗*I* *-iY*⊗*X*⊗*-iY*⊗*X*⊗*Z*⊗*I*⊗*I*
|*η*^+^⟩a_1_a_2_A_4_	|*μ*^−^⟩*b_1_b_2_B_3_*	|*ℓ^+^*⟩*c_1_c_2_c_3_C_3_*	|*ℏ*^±^⟩*D*	*X*⊗*X*⊗*-iY⊗X*⊗*X*⊗*X*⊗*X* *-iY*⊗*X*⊗*-iY*⊗*X*⊗*X*⊗*X*⊗*X*
|*η*^+^⟩a_1_a_2_A_4_	|*μ*^−^⟩*b_1_b_2_B_3_*	|*ℓ^−^*⟩*c_1_c_2_c_3_C_3_*	|*ℏ*^±^⟩*D*	*X*⊗*X*⊗*-iY*⊗*X*⊗*-iY*⊗*X*⊗*X* *-iY*⊗*X*⊗*-iY⊗X*⊗*-iY⊗X⊗X*
|*η*^−^⟩a_1_a_2_A_4_	|*ω*^+^⟩*b_1_b_2_B_3_*	|*ε*^+^⟩*c_1_c_2_c_3_C_3_*	|*ℏ*^±^⟩*D*	*-iY*⊗*X*⊗*I*⊗*I*⊗*I*⊗*I*⊗*I* *X*⊗*X*⊗*I*⊗*I*⊗*I*⊗*I*⊗*I*
|*η*^−^⟩a_1_a_2_A_4_	|*ω*^+^⟩*b_1_b_2_B_3_*	|*ε*^−^⟩*c_1_c_2_c_3_C_3_*	|*ℏ*^±^⟩*D*	*-iY*⊗*X*⊗*I*⊗*I*⊗*Z*⊗*I*⊗*I* *X*⊗*X*⊗*I*⊗*I*⊗*Z*⊗*I*⊗*I*
|*η*^−^⟩a_1_a_2_A_4_	|*ω*^+^⟩*b_1_b_2_B_3_*	|*ℓ^+^*⟩*c_1_c_2_c_3_C_3_*	|*ℏ*^±^⟩*D*	*-iY⊗X*⊗*I*⊗*I*⊗*X*⊗*X*⊗*X* *X⊗X*⊗*I*⊗*I*⊗*X*⊗*X*⊗*X*
|*η*^−^⟩a_1_a_2_A_4_	|*ω*^+^⟩*b_1_b_2_B_3_*	|*ℓ^−^*⟩*c_1_c_2_c_3_C_3_*	|*ℏ*^±^⟩*D*	*-iY*⊗*X*⊗*I*⊗*I*⊗*-iY*⊗*X*⊗*X* *X*⊗*X*⊗*I*⊗*I*⊗*-iY⊗X⊗X*
|*η*^−^⟩a_1_a_2_A_4_	|*ω*^−^⟩*b_1_b_2_B_3_*	|*ε*^+^⟩*c_1_c_2_c_3_C_3_*	|*ℏ*^±^⟩*D*	*-iY*⊗*X*⊗*Z*⊗*I*⊗*I*⊗*I*⊗*I* *X*⊗*X*⊗*Z*⊗*I*⊗*I*⊗*I*⊗*I*
|*η*^−^⟩a_1_a_2_A_4_	|*ω*^−^⟩*b_1_b_2_B_3_*	|*ε*^−^⟩*c_1_c_2_c_3_C_3_*	|*ℏ*^±^⟩*D*	*-iY*⊗*X*⊗*Z*⊗*I*⊗*Z*⊗*I*⊗*I* *X*⊗*X*⊗*Z*⊗*I*⊗*Z*⊗*I*⊗*I*
|*η*^−^⟩a_1_a_2_A_4_	|*ω*^−^⟩*b_1_b_2_B_3_*	|*ℓ^+^*⟩*c_1_c_2_c_3_C_3_*	|*ℏ*^±^⟩*D*	*-iY*⊗*X*⊗*Z*⊗*I*⊗*X*⊗*X*⊗*X* *X*⊗*X*⊗*Z*⊗*I*⊗*X*⊗*X*⊗*X*
|*η*^−^⟩a_1_a_2_A_4_	|*ω*^−^⟩*b_1_b_2_B_3_*	|*ℓ^−^*⟩*c_1_c_2_c_3_C_3_*	|*ℏ*^±^⟩*D*	*-iY*⊗*X*⊗*Z*⊗*I*⊗*-iY*⊗*X*⊗*X* *X*⊗*X*⊗*Z*⊗*I*⊗*-iY⊗X⊗X*
|*η*^−^⟩a_1_a_2_A_4_	|*μ*^+^⟩*b_1_b_2_B_3_*	|*ε*^+^⟩*c_1_c_2_c_3_C_3_*	|*ℏ*^±^⟩*D*	*-iY*⊗*X*⊗*X*⊗*X*⊗*I*⊗*I*⊗*I* *X*⊗*X*⊗*X*⊗*X*⊗*I*⊗*I*⊗*I*
|*η*^−^⟩a_1_a_2_A_4_	|*μ*^+^⟩*b_1_b_2_B_3_*	|*ε*^−^⟩*c_1_c_2_c_3_C_3_*	|*ℏ*^±^⟩*D*	*-iY*⊗*X*⊗*X*⊗*X*⊗*Z*⊗*I*⊗*I* *X*⊗*X*⊗*X*⊗*X*⊗*Z*⊗*I*⊗*I*
|*η*^−^⟩a_1_a_2_A_4_	|*μ*^+^⟩*b_1_b_2_B_3_*	|*ℓ^+^*⟩*c_1_c_2_c_3_C_3_*	|*ℏ*^±^⟩*D*	*-iY*⊗*X*⊗*X*⊗*X*⊗*X*⊗*X*⊗*X* *X*⊗*X*⊗*X*⊗*X*⊗*X*⊗*X*⊗*X*
|*η*^−^⟩a_1_a_2_A_4_	|*μ*^+^⟩*b_1_b_2_B_3_*	|*ℓ^−^*⟩*c_1_c_2_c_3_C_3_*	|*ℏ*^±^⟩*D*	*-iY*⊗*X*⊗*X*⊗*X*⊗*-iY*⊗*X*⊗*X* *X*⊗*X*⊗*X*⊗*X*⊗*-iY⊗X⊗X*
|*η*^−^⟩a_1_a_2_A_4_	|*μ*^−^⟩*b_1_b_2_B_3_*	|*ε*^+^⟩*c_1_c_2_c_3_C_3_*	|*ℏ*^±^⟩*D*	*-iY*⊗*X*⊗*-iY*⊗*X*⊗*I*⊗*I*⊗*I* *X*⊗*X*⊗*-iY*⊗*X*⊗*I*⊗*I*⊗*I*
|*η*^−^⟩a_1_a_2_A_4_	|*μ*^−^⟩*b_1_b_2_B_3_*	|*ε*^−^⟩*c_1_c_2_c_3_C_3_*	|*ℏ*^±^⟩*D*	*-iY*⊗*X*⊗*-iY*⊗*X*⊗*Z*⊗*I*⊗*I* *X*⊗*X*⊗*-iY*⊗*X*⊗*Z*⊗*I*⊗*I*
|*η*^−^⟩a_1_a_2_A_4_	|*μ*^−^⟩*b_1_b_2_B_3_*	|*ℓ^+^*⟩*c_1_c_2_c_3_C_3_*	|*ℏ*^±^⟩*D*	*-iY*⊗*X*⊗*-iY⊗X*⊗*X*⊗*X*⊗*X* *X*⊗*X*⊗*-iY*⊗*X*⊗*X*⊗*X*⊗*X*
|*η*^−^⟩a_1_a_2_A_4_	|*μ*^−^⟩*b_1_b_2_B_3_*	|*ℓ^−^*⟩*c_1_c_2_c_3_C_3_*	|*ℏ*^±^⟩*D*	*-iY*⊗*X*⊗*-iY*⊗*X*⊗*-iY*⊗*X*⊗*X* *X*⊗*X*⊗*-iY⊗X*⊗*-iY⊗X⊗X*

## References

[1] Bennett C.H., Brassard G., Crépeau C., Jozsa R., Peres A., Wootters W.K. (1993). Teleporting an Unknown Quantum State via Dual Classical and Einstein-Podolsky-Rosen Channels. Phys. Rev. Lett..

[2] Shi B.-S., Tomita A. (2002). Teleportation of an Unknown State by W State. Phys. Lett. A.

[3] Ursin R., Jennewein T., Aspelmeyer M., Kaltenbaek R., Lindenthal M., Walther P., Zeilinger A. (2004). Quantum Teleportation across the Danube. Nature.

[4] Agrawal P., Pati A. (2006). Perfect Teleportation and Superdense Coding with W-States. Phys. Rev. A.

[5] Dong L., Xiu X.M., Gao Y.J., Chi F. (2008). Quantum Secure Direct Communication Using W State. Commun. Theor. Phys..

[6] Ma S.-Y., Chen X.-B., Luo M.-X., Zhang R., Yang Y.-X. (2011). Remote Preparation of a Four-Particle Entangled Cluster-Type State. Opt. Commun..

[7] Saha D., Panigrahi P.K. (2012). N-Qubit Quantum Teleportation, Information Splitting and Superdense Coding through the Composite GHZ–Bell Channel. Quantum Inf. Process..

[8] Weedbrook C. (2013). Continuous-Variable Quantum Key Distribution with Entanglement in the Middle. Phys. Rev. A.

[9] Ramírez M.D.G., Falaye B.J., Sun G.-H., Cruz-Irisson M., Dong S.-H. (2017). Quantum Teleportation and Information Splitting via Four-Qubit Cluster State and a Bell State. Front. Phys..

[10] Zhang M., Shi S., Liu Y., Zheng Q., Wang Y. Comment on Quantum Teleportation and Information Splitting via Four-Qubit Cluster State and a Bell State. arXiv.

[11] Hou K., Bao D., Zhu C., Yang Y. (2019). Controlled Teleportation of an Arbitrary Two-Qubit Entanglement in Noises Environment. Quantum Inf. Process..

[12] Chen J., Li D., Liu M., Yang Y., Zhou Q. (2020). Quantum Controlled Teleportation of Bell State Using Seven-Qubit Entangled State. Int. J. Theor. Phys..

[13] Yan A. (2013). Bidirectional Controlled Teleportation via Six-Qubit Cluster State. Int. J. Theor. Phys..

[14] Kazemikhah P., Tabalvandani M.B., Mafi Y., Aghababa H. (2022). Asymmetric Bidirectional Controlled Quantum Teleportation Using Eight Qubit Cluster State. Int. J. Theor. Phys..

[15] Zha X.-W., Zou Z.-C., Qi J.-X., Song H.-Y. (2013). Bidirectional Quantum Controlled Teleportation via Five-Qubit Cluster State. Int. J. Theor. Phys..

[16] Duan Y.-J., Zha X.-W. (2014). Bidirectional Quantum Controlled Teleportation via a Six-Qubit Entangled State. Int. J. Theor. Phys..

[17] Wang X., Mo Z. (2017). Bidirectional Controlled Joint Remote State Preparation via a Seven-Qubit Entangled State. Int. J. Theor. Phys..

[18] Shi J., Zhan Y.-B. (2018). Scheme for Asymmetric and Deterministic Controlled Bidirectional Joint Remote State Preparation. Commun. Theor. Phys..

[19] Li Y., Qiao Y., Sang M., Nie Y. (2019). Bidirectional Controlled Remote State Preparation of an Arbitrary Two-Qubit State. Int. J. Theor. Phys..

[20] Ma P.-C., Chen G.-B., Li X.-W., Zhan Y.-B. (2018). Schemes for Hybrid Bidirectional Controlled Quantum Communication via Multi-Qubit Entangled States. Int. J. Theor. Phys..

[21] Chen Y.-X., Du J., Liu S.-Y., Wang X.-H. (2017). Cyclic Quantum Teleportation. Quantum Inf. Process..

[22] Sang Z. (2018). Cyclic Controlled Teleportation by Using a Seven-Qubit Entangled State. Int. J. Theor. Phys..

[23] Wang M., Yang C., Mousoli R. (1970). Controlled Cyclic Remote State Preparation of Arbitrary Qubit States. CMC Comput. Mater. Contin..

[24] Sun S., Zhang H. (2020). Quantum Double-Direction Cyclic Controlled Communication via a Thirteen-Qubit Entangled State. Quantum Inf. Process..

[25] Zhang D., Zha X.-W., Duan Y.-J. (2015). Bidirectional and asymmetric quantum controlled teleportation. Int. J. Theor. Phys..

[26] Choudhury B.S., Samanta S. (2017). Asymmetric bidirectional 3 ⇔ 2 qubit teleportation protocol between Alice and Bob via 9-qubit cluster state. Int. J. Theor. Phys..

[27] Jie X., Zhou R.-G. (2023). Asymmetric bidirectional cyclic controlled quantum teleportation in noisy environment. Quantum Inf. Process..

[28] Huo G., Zhang T., Zha X., Zhang X., Zhang M. (2021). Controlled asymmetric bidirectional quantum teleportation of two-and three-qubit states. Quantum Inf. Process..

[29] Oh S., Lee S., Lee H.W. (2002). Fidelity of quantum teleportation through noisy channels. Phys. Rev. A.

[30] Jung E., Hwang M.R., Ju Y.H., Kim M.S., Yoo S.K., Kim H., Park D., Son J.-W., Tamaryan S., Cha S.K. (2008). Greenberger-Horne-Zeilinger versus W states: Quantum teleportation through noisy channels. Phys. Rev. A—At. Mol. Opt. Phys..

[31] Hu M.L. (2011). Environment-induced decay of teleportation fidelity of the one-qubit state. Phys. Lett. A.

[32] Hu M.-L. (2012). Disentanglement, Bell-nonlocality violation and teleportation capacity of the decaying tripartite states. Ann. Phys..

[33] Li Y.-L., Zu C.-J., Wei D.-M. (2019). Enhance quantum teleportation under correlated amplitude damping decoherence by weak measurement and quantum measurement reversal. Quantum Inf. Process..

[34] Liang X.-T. (2003). Classical Information Capacities of Some Single Qubit Quantum Noisy Channels. Commun. Theor. Phys..

[35] Gao T., Yan F.L., Wang Z.X. (2005). Controlled Quantum Teleportation and Secure Direct Communication. Chin. Phys..

[36] Sang M. (2016). Bidirectional Quantum Controlled Teleportation by Using a Seven-Qubit Entangled State. Int. J. Theor. Phys..

[37] Shao Z., Long Y. (2019). Circular Controlled Quantum Teleportation by a Genuine Seven-Qubit Entangled State. Int. J. Theor. Phys..

[38] Jiang Y.-L., Zhou R.-G., Hao D.-Y., Hu W. (2021). Bidirectional Controlled Quantum Teleportation of Three-Qubit State by a New Entangled Eleven-Qubit State. Int. J. Theor. Phys..

[39] Zhou R.-G., Qian C., Ian H. (2019). Cyclic and Bidirectional Quantum Teleportation via Pseudo Multi-Qubit States. IEEE Access.

[40] Jiang S.-X., Zhou R.-G., Xu R., Luo G. (2019). Cyclic Hybrid Double-Channel Quantum Communication via Bell-State and GHZ-State in Noisy Environments. IEEE Access.

